# Biosynthesis of Antifungal Solanimycin May Involve an Iterative Nonribosomal Peptide Synthetase Module

**DOI:** 10.1021/acschembio.2c00947

**Published:** 2023-04-17

**Authors:** Annabel C. Murphy, Matthew Corney, Rita E. Monson, Miguel A. Matilla, George P. C. Salmond, Finian J. Leeper

**Affiliations:** † Yusuf Hamied Department of Chemistry, University of Cambridge, Lensfield Road, Cambridge CB2 1EW, U.K.; ‡ Department of Biochemistry, University of Cambridge, Tennis Court Road, Cambridge CB2 1QW, U.K.

## Abstract

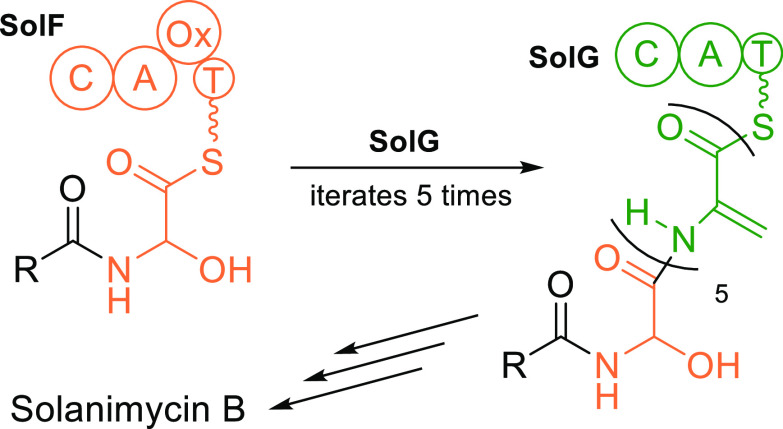

*Dickeya solani*, a plant-pathogenic bacterium, produces solanimycin, a potent hybrid polyketide/nonribosomal peptide (PKS/NRPS) anti-fungal compound. The biosynthetic gene cluster responsible for synthesis of this compound has been identified. Because of instability, the complete structure of the compound has not yet been elucidated, but LC-MS^2^ identified that the cluster produces two main compounds, solanimycin A and B, differing by a single hydroxyl group. The fragmentation pattern revealed that the central part of solanimycin A is a hexapeptide, Gly-Dha-Dha-Dha-Dha-Dha (where Dha is dehydroalanine). This is supported by isotopic labeling studies using labeled serine and glycine. The N-terminal group is a polyketide-derived C_16_ acyl group containing a conjugated hexaene, a hydroxyl, and an amino group. The additional hydroxyl group in solanimycin B is on the α-carbon of the glycine residue. The incorporation of five sequential Dha residues is unprecedented because there is only one NRPS module in the cluster that is predicted to activate and attach serine (which is subsequently dehydrated to Dha), meaning that this NRPS module must act iteratively. While a few other iterative NRPS modules are known, they all involve iteration of two or three modules. We believe that the repetitive use of a *single* module makes the solanimycin biosynthetic pathway unique among NRPSs so far reported.

## Introduction

Global human population continues to rise and may be around 10 billion by the middle of the century. Global demand for food will continue to rise, so food security is of profound importance^1^ to our collective future if we are to avoid widespread famines. Crop protection is crucial in the food production chain and some recent cogent reviews^1−5^ have highlighted the growing, and potentially devastating, importance of fungal diseases in crop economics, environmental impacts, nutrition, health, and longevity. With fungicides, as with antibiotics against bacteria, development of resistance is a serious problem.^4,5^ In order to sustain a healthy growing global population, further discoveries in the plant protection area are required^2−5^ and in the antifungal arena in particular.

**Figure 1:**
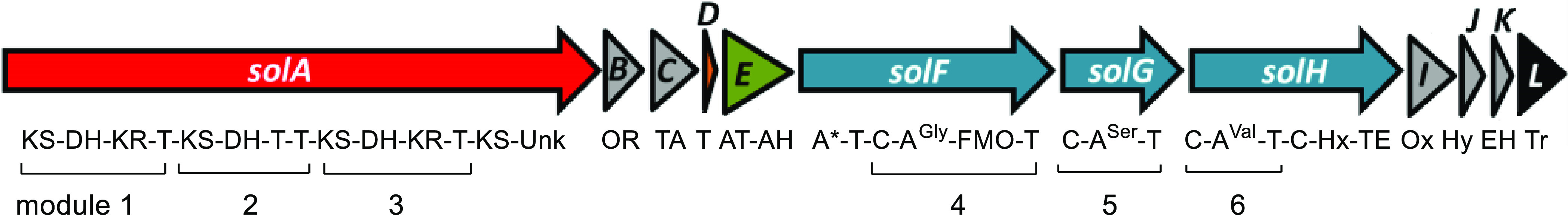
Gene cluster that produces solanimycin: red = polyketide synthase (PKS), blue = nonribosomal peptide synthase (NRPS), green = *trans*-acyl transferase (AT); KS = ketosynthase, DH = dehydratase, KR = ketoreductase, T = thiolation domain (acyl carrier protein or peptidyl carrier protein), Unk = domain of unknown function found in NRPSs, Hx = HxxPF-repeat domain (unknown function found in NRPSs), OR = NAD(P)-dependent oxidoreductase, TA = PLP-dependent transaminase (aminotransferase), AH = acyl hydrolase, C = NRPS condensation domain, A^Gly^ = adenylation (A) domain predicted to activate Gly, A* = A domain with unknown substrate, FMO = flavin mono-oxygenase, Ox = cytochrome P450, Hy = Zn-dependent hydrolase, EH = enoyl CoA hydratase, and Tr = MATE efflux transporter.

Plant environments are highly competitive niches, and plant-associated microorganisms very often produce antibiotics for competitive fitness.^6,7^ For example, we published the initial studies on the biosynthesis of oocydin A,^8,9^ an anti-oomycete and antifungal chlorinated polyketide macrolide made by various strains of *Serratia* and *Dickeya*, and others have subsequently clarified various aspects of the biosynthesis.^10,11^ During our studies on oocydin A in the bacterium *Dickeya solani* MK10, we noticed that transposon insertion mutants defective in oocydin A production (*ooc^–^*) retained antifungal activity.^13^*Dickeya solani* is a plant pathogen currently causing severe crop losses on continental Europe.^12^ There is a complete genome sequence, without gaps, available for strain MK10 (GenBank GCA_000365285.1). Further transposon mutagenesis studies on *ooc^–^* strains of *D. solani* showed that this strong residual antifungal activity was abolished by transposon insertions in just one gene cluster coding for an unknown natural product named solanimycin.^13^ The organization of the cluster and the annotation of the genes and their subdomains based on the gene sequence are shown in Figure 1. The cluster features one polyketide synthase (PKS) and three nonribosomal peptide synthetase (NRPS) genes, suggesting a product of mixed origin. There are also genes coding for a *trans*-acyl transferase (AT), a standalone acyl carrier protein/thiolation domain (T), five tailoring enzymes, and an efflux transporter. In-frame deletion mutants were created for each of the genes (*solA-L*) and most of the mutants had no antifungal activity (*solA-D* and *F-I*) and two had very much reduced activity (*solE* and *K*).^13^

**Figure 2:**
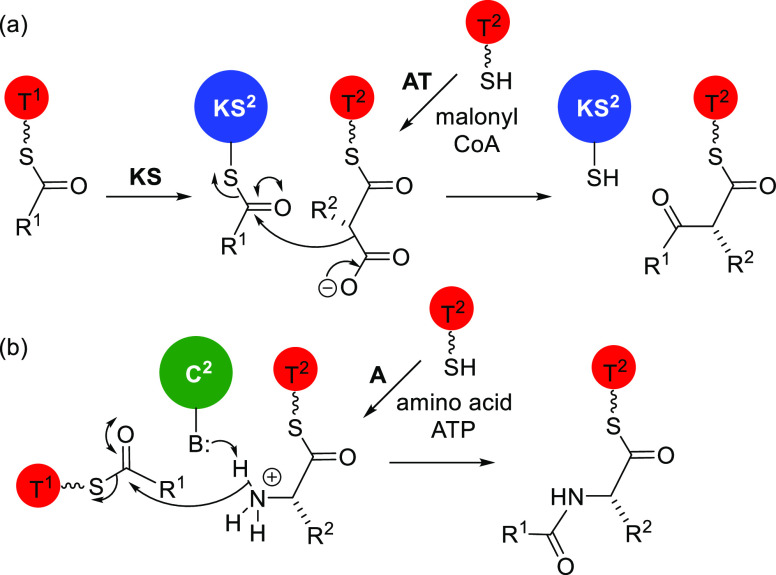
Comparison of the chain-extension reaction in (a) PKSs and (b) NRPSs. The key difference is that in PKSs, the donor acyl group is transferred to the KS, where it can be held, whereas in NRPSs, it is transferred directly to the acyl acceptor.

PKSs and NRPSs are both multi-domain enzymes that share a common thioester-templated biosynthetic mechanism where intermediates on the pathway are tethered to and translocated across the modular enzyme throughout the biosynthesis.^14−17^ In both cases, small monomers are selected (by ATs in PKSs or adenylation domains, A, in NRPSs) and transferred to a phosphopantetheine cofactor attached to T domains forming thioesters. Nucleophilic attack of monomers attached to the downstream T domain is catalyzed by a ketosynthase (KS) to form a C–C bond in PKSs or a condensation domain (C) to form an amide bond in NRPSs (Figure 2). A range of further chemical changes can be catalyzed, usually by additional domains within the PKS or NRPS, prior to further rounds of extension, until the full-length natural product backbone is formed. At this point, the tethered product can be released often via a thioesterase domain (TE) that may catalyze hydrolysis or macrocyclization. Often, the multidomain architecture of PKS/NRPS enzymes combined with bioinformatic tools that can predict the activated monomer incorporated by each module allows prediction of the structure of an unknown natural product via bioinformatic analysis of the gene cluster responsible for its biosynthesis. Such an analysis was employed to tentatively propose a structure for solanimycin.^13^ Not all PKS or NRPS systems strictly follow this colinearity between the domain order and product structure, however: iterative PKSs are common and a few iterative (nonlinear) NRPSs are known (see later Discussion).^17^

In this study, we report our studies on the structure and biosynthesis of the novel antifungal compound, solanimycin, and our discovery that this cluster features highly unusual iterative use of a single NRPS module.

## Results

### Isolation and Partial Purification

Antifungal activity was found in the culture medium of *Dickeya solani* MK10 *ooc*^–^ cultures. Direct extraction of the active component into organic solvents was attempted but no activity was extracted into ethyl acetate (with or without adjustment of the medium to acidic or basic pH) and extraction with butan-1-ol or dichloromethane–isopropanol (4:1) also left most of the activity in the aqueous layer. As a result, the supernatant was freeze-dried, and the residue was extracted with organic solvents. Only methanol gave a good level of extraction of the antifungal activity. Optimization of the levels of potato dextrose in the growth medium to 5 g/L facilitated downstream processing by reducing the levels of media components extracted.

This extract was then concentrated and purified successively on gravity-flow columns of LH20 and C_18_ silica to give semi-purified solanimycin (5–10 mg/L supernatant). Elution of solanimycin from the C_18_ column required 70% acetonitrile in water, indicative of a moderately hydrophobic compound, which was surprising given the difficulties encountered with extraction into organic solvents. Many attempts were made to further purify the solanimycin on reverse-phase high-performance liquid chromatography (HPLC), identifying fractions by bioassay, but the recovered mass and antifungal activity were always much less than that had been put on the column, and the purity (judged by μg of extract required to detect the antifungal activity) had not improved.

### Mass Spectrometry

In view of the instability of more highly purified extracts, we had to deduce as much as we could by comparison of the spectral data for the semi-purified solanimycin with those of an identically purified sample from a mutant (*sol^–^*) strain in which production of solanimycin had been abolished by transposon insertion in the first gene of the biosynthetic gene cluster. Comparison of liquid chromatography–mass spectrometry (LC–MS) traces for extracts of the *sol*^+^ and *sol^–^* strains revealed the presence of two overlapping peaks with accurate masses of 963.480 and 979.474 (M + H^+^ ions) in the *sol^+^* but not the *sol^–^* strain (Figures S1 and S2). XCMS metabolomics comparison^18^ of the LC–MS data did not reveal any further candidate ions (other than alternative adducts, e.g., M + Na^+^, of these two). The difference in masses corresponds to one oxygen atom. We have named the two compounds solanimycin A (963.480) and solanimycin B (979.474).

**Figure 3:**
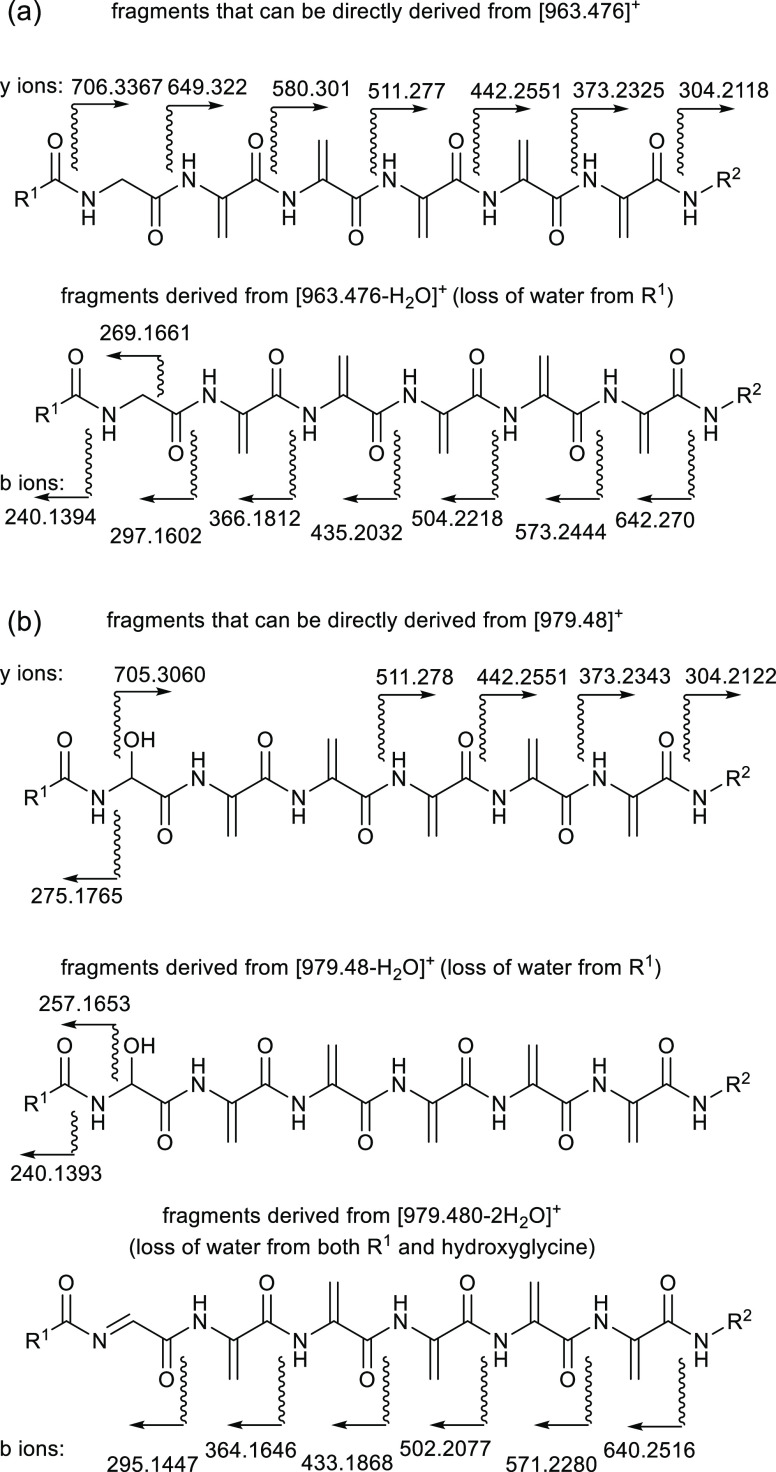
Observed fragments in MS–MS for (a) solanimycin A and (b) solanimycin B.

MS–MS with collision-induced dissociation on these ions was very informative (Figure S3). Both ions readily lose water and solanimycin B also loses a second water, consistent with it having an extra oxygen atom. Many of the smaller fragments appear to be derived from these dehydrated ions. The first prominent fragment ion with a mass less than these dehydration products is at 706.3367 for solanimycin A and at 705.3060 for solanimycin B. The difference in these two masses corresponds to the difference between NH_3_ in the former and O in the latter. We interpret this in terms of the different fragmentations expected for a normal glycine residue and one that has been hydroxylated on the α-carbon (Figure 3a,b). This is consistent with the presence of an NRPS module (module 4 in SolF) which is predicted to activate glycine and contains a predicted flavin mono-oxygenase domain.

Most of the remaining fragment ions appear to be the b and y ions expected for fragmentation of a peptide. Intriguingly, most of the intervals between fragments in both the b and y series and in both compounds are close to 69.022, which corresponds to C_3_H_3_NO, fitting with dehydroalanine (Dha). Complete analysis of the MS–MS spectrum indicated that the glycine or hydroxyglycine residue is followed by a string of five dehydroalanine residues. Evidence is given below that these are dehydro-α-alanine residues and not dehydro-β-alanine. This hexapeptide has additional groups on the N- and C-termini (R^1^ and R^2^), for which there was very little information from MS other than their masses. Figure 3 gives the structure and fragmentation data for the central hexapeptide portion of both compounds.

### Identity of the N-Terminal Group R_1_

**Figure 4:**
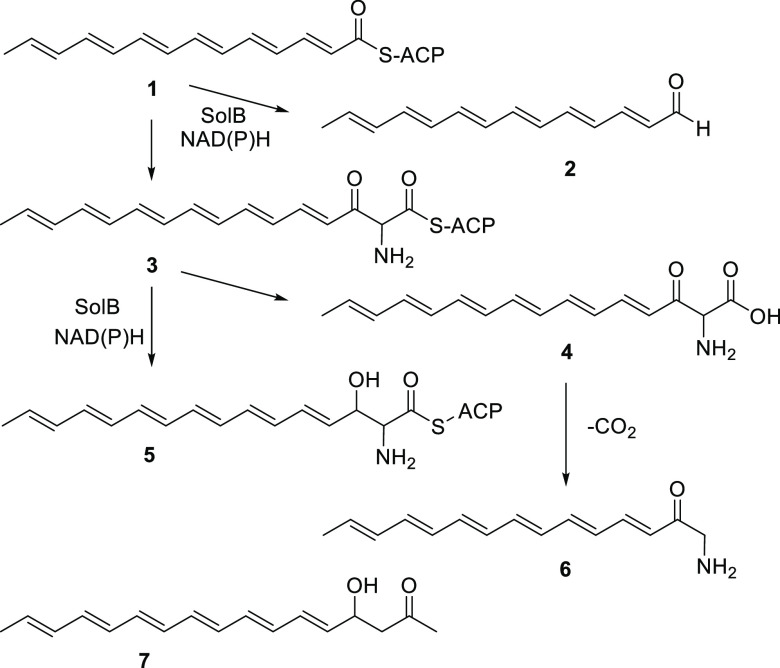
Proposed formation of shunt metabolites **2** and **6**. The acyl group of **5** is proposed as the N-terminal group (R_1_CO) of solanimycin. **7** is a shunt metabolite from an enediyne PKS, which has the same absorption spectrum as solanimycin.^20^

Clues as to the identity of the N-terminal group came from metabolites isolated from strains in which individual genes had been knocked out. In-frame deletion of *solF* (encoding an NRPS protein) resulted in production of an orange cell-associated pigment. This was extracted into ethanol and purified by HPLC. ^1^H NMR showed that the compound was not completely pure, being contaminated with some fatty materials with peaks in the 0.9–1.5 ppm region, but further purification was not attempted due to its instability. Using two-dimensional COSY and HSQC spectra (Figures S4–S6), the pigment was readily assigned as the polyene aldehyde tetradeca-2,4,6,8,10,12-hexaenal **2** (Figure 4). The coupling constants which could be measured were in the range 11–15 Hz, so we assume that all the double bonds are *trans* but, due to peak overlap, not every coupling constant could be measured. The UV–visible spectrum (single broad peak at 390 nm) matched the literature value^19^ and the accurate mass (using ASAP ionization) was correct also (Figures S7 and S8). This aldehyde has presumably been formed by enzymic reduction of the corresponding acyl carrier protein (ACP) thioester **1**. The only gene encoding a likely reductase in the cluster is *solB*.

In-frame deletion of *solB* (a predicted NAD(P)-dependent oxidoreductase) also resulted in production of a yellow-orange cell-associated pigment, but this pigment was more polar than aldehyde **2**. The UV–visible spectrum of the pigment was very similar to **2** (broad peak at 385 nm, Figure S9) and comparison of the extract’s LC–MS with that of the equivalent extract from the *sol^–^* strain showed the additional presence of two closely spaced peaks both showing *m*/*z* 230.154 (M + H^+^) (Figure S10). The major pigment peak was partially purified by HPLC and, although still very impure, it was not purified further due to the low amount of material and the instability of the pigment. Nevertheless, the 5.5–7.5 ppm region of the ^1^H NMR spectrum (Figure S11) showed a series of peaks very similar to (but slightly shifted from) the peaks for the olefinic carbons of **2**. In particular, the double quartet for the olefinic proton adjacent to the terminal methyl group was clearly visible at the same chemical shift. It is clear from this and from the UV–visible spectrum that the tetradeca-2,4,6,8,10,12-hexaenoyl chromophore is still present. The extra molecular mass corresponds to an additional CH_3_N compared to **1**. We believe that the only structure that fits the data and is consistent with the pigment being a shunt metabolite from the pathway to solanimycin is the aminomethyl ketone **6**. This could be formed by spontaneous decarboxylation of acid **4**, formed by hydrolysis of the corresponding ACP thioester **3**.

It is notable that reduction of the keto group of **3** to the alcohol **5** (presumably by SolB) would give an acyl group that exactly matches the mass of the N-terminal group of solanimycin. Further evidence that this is the N-terminal group of solanimycin came from the UV–visible spectrum. Compared to the equivalent extract from the *sol^–^* strain, semi-purified solanimycin shows five absorption maxima in the 300–400 nm region with the largest two being at 358 and 379 nm (Figure S12). This exactly matches the absorption spectrum expected for a linear hexaene not conjugated to a carbonyl group. For example, β-hydroxyketone **7**, which is a shunt metabolite produced by an enediyne PKS, has a nearly identical set of five peaks in this region.^20^

In addition to the *solB* and *solF* mutants described above, the *solC*, *I*, and *K* knockout mutants were studied using XCMS metabolomics,^18^ but no new compounds were observed to accumulate in quantities that would allow isolation and structure determination.

### NMR Spectroscopy

Two-dimensional NMR spectra of the semi-purified solanimycin (Figures S13–S16) confirmed the presence of both the multiple dehydroalanine residues and the polyene. Phase-sensitive HSQC showed a collection of peaks at 5.7–6.1 ppm in the ^1^H NMR and 110–115 ppm in the ^13^C NMR which were not present in the equivalently purified extract from the *sol^–^* strain. These are the chemical shifts expected for dehydroalanine residues^21,22^ and, in confirmation, the phase of the peaks indicated that they arose from CH_2_ groups (not C, CH, or CH_3_). In the HMBC spectrum, these ^1^H peaks correlated to ^13^C atoms at ca. 135 and 165 ppm, as expected for the α and carbonyl carbons, respectively, of dehydroalanine residues. Also visible in the HSQC spectrum are a collection of CH peaks at 6.2–6.4 in the ^1^H NMR and 130–135 ppm in the ^13^C NMR which do not appear in the *sol*^–^ sample. These are as expected for the polyene portion of solanimycin. In the HMBC spectrum, ^1^H peaks in this region correlated to ^13^C atoms at ca. 19 and 72 ppm, as expected for the terminal methyl group and the 3-CHOH carbons, respectively, of the proposed N-terminal acyl group **5**. Unfortunately, any further analysis of the NMR spectra was not possible due to the high level of impurities present that gave large peaks especially in the 0.8–2.5 and 3.1–4.0 ppm regions of the ^1^H NMR spectrum.

### Isotopic Labeling Experiments

In order to get further confirmation of the peptidic central portion of solanimycin, isotopic labeling experiments were undertaken to confirm that the precursors of the different residues were as expected.

Dehydroalanine is normally derived by dehydration of serine, in both NRPS^23,24^ and ribosomally derived peptides (RiPPs),^25^ so the solanimycin-producing strain was grown in the presence of [3,3-^2^H_2_]serine (2 and 8 mM). The LC–MS spectra of the solanimycins from these experiments showed that at the lower concentration, the level of incorporation was significant, with a clear increase in the size of the [M + H + 2] peak, but at the higher concentration, far higher incorporation was seen. The isotope distribution pattern in the latter case was bimodal with one set of peaks centered around [M + H + 2] to [M + H + 4] and another (lower) set of peaks centered around [M + H + 11] to [M + H + 14] (Figure S17). The same distribution was seen for both solanimycin A (M + H at 963) and solanimycin B (M + H at 979) but the ratio A:B (normally ca. 60:40) was higher in these experiments (ca. 85:15).

**Figure 5:**
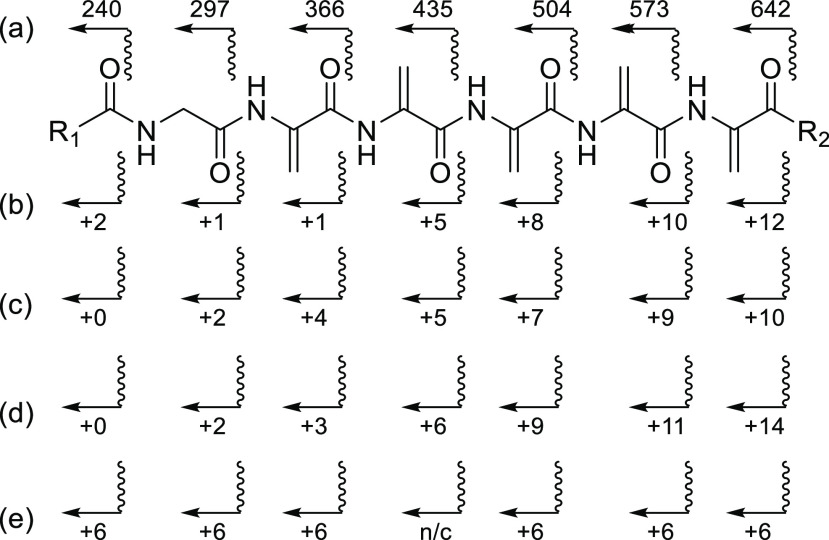
Observed fragments (b ions only) in MS–MS of solanimycin A (a) unlabeled (M + H ion selected for fragmentation); (b–e) increments observed for each fragment for isotopically labeled samples produced by incorporation of (b) [3,3-^2^H_2_]serine (M + H + 14 ion selected), (c) [2,2-^2^H_2_]glycine (M + H + 11 ion selected), (d) [1,2-^13^C_2_]glycine (M + H + 14 ion selected), and (e) sodium [1,2-^13^C_2_]acetate (M + H + 12 ion selected). “n/c” indicates not clear.

MS–MS fragmentation was performed on the peaks centered at [M + H + 14] for solanimycin A to determine the distribution of the incorporated deuterium atoms (Figure S18). Figure 5b shows the average number of deuterium atoms incorporated in each b ion derived from these parent ions. Although not entirely consistent (probably due to small amounts of other ions at the same masses), it is apparent that there are on average ca. 1.5 to 2 deuterium atoms in each dehydroalanine residue and none in the glycine, while the end groups (R^1^ and R^2^) have very little deuterium (1 to 2 at most). Due to the low level of solanimycin B produced in this experiment, clear fragmentation of its selected ions could not be observed.

Next, [2,2-^2^H_2_]glycine (2 and 10 mM) was used to confirm its incorporation into the glycyl residue of solanimycin. The level of deuterium incorporation was far higher than that would be possible if only the glycyl residue was labeled. Even at 2 mM, the average number of deuterium atoms incorporated was 7, and at 10 mM, it was 8–9 (Figure S19). Again, the ratio of solanimycin A to B was much higher than usual. The distribution of deuterium was determined for solanimycin A by MS–MS fragmentation of the ions centered on the [M + H + 11] ion (Figures 5c, S20 and S21). Again, this showed ca. 1.5–2 deuterium atoms in each dehydroalanine residue as well as, in this experiment, the same level in the glycyl residue. Almost no labeling of the end groups was observed.

The labeling of the dehydroalanine residues can be explained by the well-established glycine cleavage system^26^ in which glycine is oxidatively decarboxylated and deaminated to give formaldehyde covalently attached to tetrahydrofolate (THF) as methylene THF. This is the methylene donor in a reaction in which glycine is hydroxymethylated to give serine. A dehydroalanine residue resulting from this pathway would retain two deuterium atoms in its methylene group. This pathway must be very active in *D. solani* even at 2 mM of administered glycine.

With [1,2-^13^C_2_]glycine (2 mM), even higher levels of incorporation were observed. This is because serine made from two molecules of [1,2-^13^C_2_]glycine would have three ^13^C atoms. The resulting solanimycin A and B both had an average of 10 ^13^C atoms with some molecules having up to 15 ^13^C atoms (Figure S19D). Fragmentation was performed on the solanimycin A ions centered at M + H + 14 (Figure 5d) and showed that each dehydroalanine residue had 2–3 ^13^C atoms and the glycine residue had 2 ^13^C atoms (Figures S20 and S21). Again, the levels of solanimycin B produced were sufficiently low that no useful fragmentation data could be obtained for that compound. Sufficient sample was obtained in this case for NMR spectroscopy on the semi-purified solanimycin extract to confirm the location of the ^13^C atoms. A two-dimensional ^13^C–^13^C COSY spectrum (Figure S22) revealed off-diagonal peaks for the coupling between the α- and β-carbons and between the α-carbons and the carbonyl carbons of the dehydroalanine residues and also between the α-carbon and the carbonyl carbon of the glycine residue. In the HSQC spectrum, the doublet nature of the carbon signals for the CH_2_ groups of the dehydroalanine residues (due to ^13^C–^13^C coupling) could be clearly seen (Figure S23).

As the last module of the NRPS (SolH) is predicted to incorporate valine, we next fed *d*_8_-valine (5 mM) to the producing strain. Strong incorporation of *d*_8_-valine was observed with the most prominent peaks being at *m*/*z* 971 and 987, eight mass units higher than unlabeled solanimycins A and B, respectively (Figure S24). In the MS–MS fragmentation on *m*/*z* 971, this eight-unit mass shift was seen in all the y ions (which retain the C-terminus) but none of the b ions (which retain the N-terminus), thus showing that the eight deuterium atoms are all in the C-terminal unit (Figure S25).

Lastly, as the N-terminal acyl group is predicted to be polyketide-derived, sodium [1,2-^13^C_2_]acetate was fed to the organism. At 2 mM, there was hardly any incorporation; at 5 mM, there were visible increases in the M + H + 1 and M + H + 2 peaks, but at 20 mM, more than half the solanimycin A molecules showed heavy ^13^C labeling, peaking around M + H + 11 (Figure S26). Fragmentation of ions centered at M + H + 12 (Figures 5e and S27) revealed that there was no labeling of the dehydroalanine or glycine residues and half of the 12 ^13^C atoms were located in the N-terminal acyl group (R^1^-C=O in Figure 3) and the other half were in the C-terminal group (R^2^), suggesting that this too is polyketide or fatty acid-derived. (Isoprenoid via the mevalonate pathway would also be possible but *D. solani* uses the nonmevalonate pathway.)

## Discussion

### Structure of Solanimycins A and B

**Figure 6:**
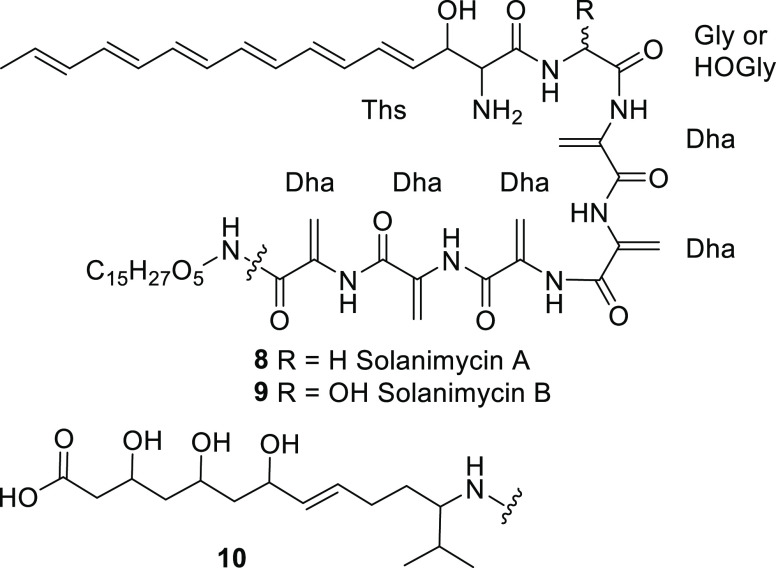
Structure of solanimycin A **8** and B **9** and an illustrative possible structure **10** for the C-terminal group. Ths = β-(trideca-1,3,5,7,9,11-hexaenyl)serine.

The structures of the central hexapeptide units of solanimycins A and B are firmly established from the MS–MS spectra of the LC–MS peaks which appear only in the *sol*^+^ extract and not in the *sol^–^* extract. This is corroborated both by the observation of peaks at the correct chemical shift in the ^1^H and ^13^C NMR spectra of semi-purified solanimycin and by the labeling experiments with isotopically labeled serine and glycine. The structure of the N-terminal group comes largely from the isolation of shunt metabolites **2** and **6** from Δ*solG* and Δ*solB* knockout strains, respectively. This is backed up by the appearance of peaks at the correct chemical shift in the ^1^H and ^13^C NMR spectra of semi-purified solanimycin and by its UV–visible spectrum which shows the linear hexaene chromophore. Thus, the structures of solanimycins A and B, as far as they can be deduced, are **8** and **9** (Figure 6).

There are few clues as to the identity of the C-terminal group other than the measured mass of the fragment, *m*/*z* 304.2122 (for R^2^-NH_3_^+^) and the fact that valine is incorporated. However, this accurate mass only gives three possible molecular formulae within 5 p.p.m. (assuming only C, H, N, O and S): C_15_H_30_NO_5_^+^ (calc. 304.2118), C_16_H_34_NS_2_^+^ (calc. 304.2127), and C_16_H_26_N_5_O (calc. 304.2132). Given that the incorporation of sodium [1,2-^13^C_2_]acetate showed that this group is largely acetate-derived, the first (and closest) of these seems the only likely formula. It is notable that incorporation of one valine followed by a polyketide chain extension could give a structure such as **10** as the C-terminal group of solanimycin and this has exactly the proposed molecular formula (although many other arrangements of the functional groups would be possible and equally likely).

From the integration of the ^1^H NMR spectrum of the semipurified solanimycin, it could be estimated that only about 5% of the mixture consisted of solanimycin A or B. This equates to about 0.25–0.5 mg per liter of the culture medium (0.26–0.52 μM). In our earlier study,^13^ we showed that solanimycin at 0.6 times this concentration (0.16–0.31 μM) stopped the growth of *Schizosaccharomyces pombe* and caused a 542-fold reduction in viable colonies over 3 h compared to the solvent control. It is, therefore, a very potent antifungal agent.

Several aspects of the structure of the solanimycins are unusual. First, while the methyl-terminated hexaene chain has been observed in the myxochromides made by *Myxococcus* and *Stigmatella* strains^27−30^ (which are in fact hepta- or octa-enes), we are not aware of any other examples. Such polyene chains are more often encountered as precursors of metabolites where the polyene chain has undergone cyclization reactions, e.g., the enediynes,^20^ polycyclic tetronate macrolactams,^31^ and serpentenes.^32^

Second, the α-hydroxyglycyl residue of solanimycin B is very unusual. A number of examples of α-hydroxylated amino acid residues are known, especially among diketopiperazines (DKPs). The most well-known example is bicyclomycin, a clinically used broad-spectrum antibiotic.^33^ Some DKPs are hydroxylated on both α-positions, e.g., lepistamide B.^34^ There is one report of hydroxyglycine in a naturally occurring DKP and that is the very simple 3-hydroxy-2,5-diketopiperazine.^35^ However, the cyclic structure of DKPs helps stabilize the carbinolamide from spontaneous cleavage into aldehyde and amide. Among other peptidic natural products, an α-hydroxyglycyl unit is found in macrocyclic depsipeptides skyllamycin A (RP-1776)^36^ and maltacine D1a.^37^ However, the only acyclic α-hydroxyglycine residue that we have found is in spergualin, isolated in 1981 from *Bacillus laterosporus* BMG162-aF2.^38^ A deoxy derivative of spergualin (although still containing the α-hydroxyglycine) is a clinically used immunosuppressive drug called gusperimus.^39^

Perhaps the most unusual feature is the run of five consecutive dehydroalanine residues in solanimycins A and B. Dehydroalanine residues are found in ribosomally as well as nonribosomally produced peptides, but more than one dehydroalanine residue in a row is rare. Most such examples are in the macrocyclic thiopeptide (or thiazolyl peptide) family, e.g., thiostrepton (the best known thiopeptide) has an acyclic C-terminal tail ending in two dehydroalanines,^40^ sulfomycin I^41^ has three and six thiopeptides^42−45^ have four. Lantibiotic lichenicidin β contains three dehydroalanine residues in a row.^46^ However, we have been unable to find any natural product with five dehydroalanine residues in a row. Furthermore, all the examples that have more than one dehydroalanine in a row have been ribosomally derived peptides, so the solanimycins, being NRPS-derived, are especially unusual. Recently, a nonribosomal tripeptide has been reported that has dehydroalanine adjacent to dehydrobutyrine.^23^

### Proposed Biosynthesis of Solanimycins A and B

**Figure 7:**
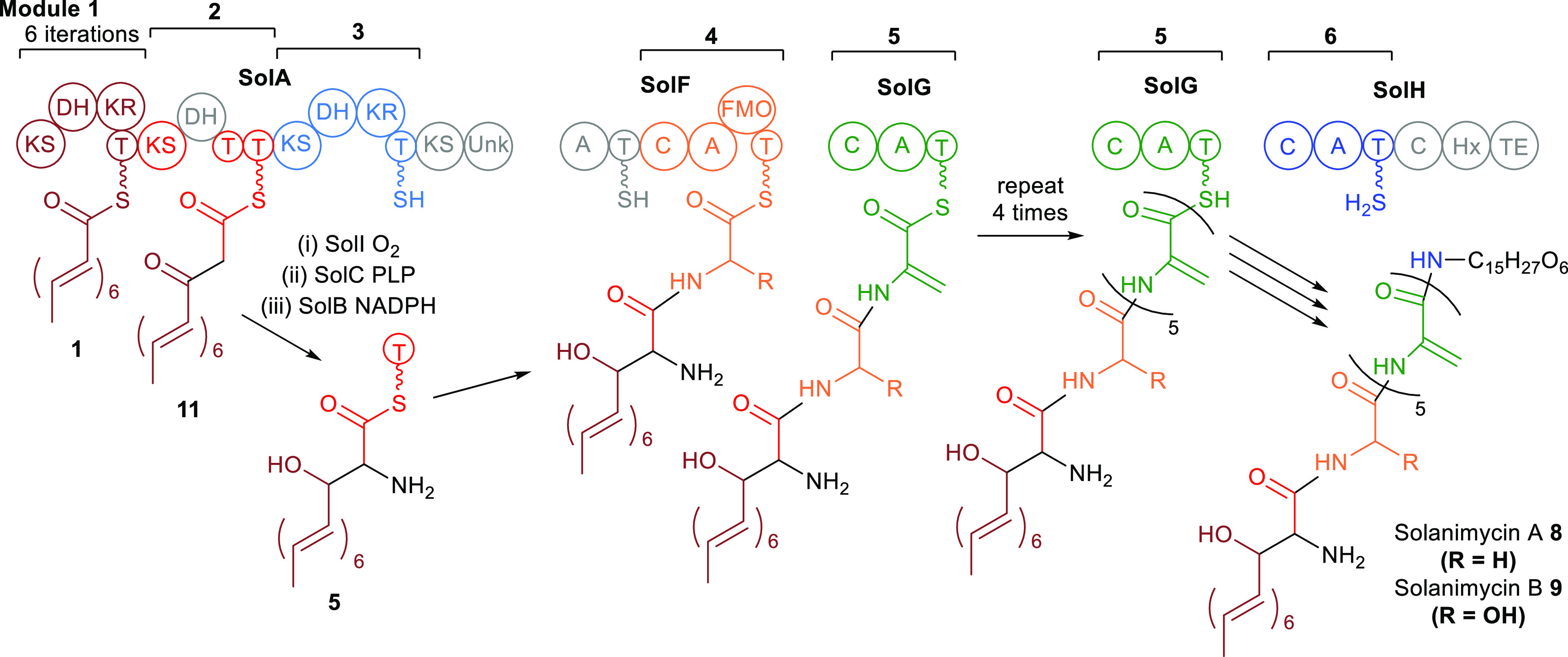
Proposed biosynthesis of solanimycins A (**8**, R = H) and B (**9**, R = OH). For **8**, the hydroxylation step catalyzed by the FMO domain in module 4 is skipped. See Figure 1 for domain abbreviations.

The gene cluster for the solanimycins only has one polyketide synthase (PKS) gene, *solA*, and the encoded PKS consists of only three modules (plus a lone KS domain at the end). All the modules lack *cis*-AT domains and the *trans*-AT domain is in SolE along with a second AT-like domain whose sequence suggests that it is the proof-reading acyl hydrolase, commonly found in *trans*-AT PKS clusters.^14^ There are not enough modules for the N-terminal acyl group to be produced in a processive fashion, so we propose that module 1 (or possibly module 3) is iterative and produces the polyene portion of this acyl group (both have the required KR and DH domains) (Figure 7). Alternatively, a polyunsaturated acyl group might be synthesized by an iterative PKS encoded elsewhere in the genome, and only the final elongation steps are catalyzed by SolA. In the *solF* knockout strain, the PKS production line probably gets stalled because of the lack of SolF, which accepts the acyl group further down the line, and under these circumstances, a prominent release mechanism is reduction (presumably by SolB) of the C_14_ polyunsaturated thioester **1** to give the observed aldehyde **2** (Figure 4).

The thioester **1** only needs two additional carbons and an attached amino group to complete the C-terminal acyl group. This could possibly have come from glycine, but our feeding experiments with ^2^H- and ^13^C-labeled glycine show that it does not. It could also have come from an aminomalonyl chain-extension unit, but the genes required for biosynthesis of aminomalonyl CoA are not present in the cluster. Instead, we propose that the second module of the PKS, which lacks a ketoreductase (KR) domain, simply catalyzes a single round of chain extension to give β-keto-thioester **11** (Figure 7). This is then oxidized at C-2 (by P450 SolI) to a keto group, which is then transaminated to the amine **3** by PLP-dependent SolC. Similar conversion of a polyketide chain into a nonproteinogenic α-amino acid by the actions of a P450 and a transaminase is involved in the biosynthesis of cyclosporin A^47^ and the oxaleimides.^48^ Finally, reduction of the β-keto-group by SolB gives the C-terminal acyl group **5** ready to be passed on to the NRPS. In the absence of SolB, reduction of the β-keto-group does not occur and slow hydrolytic cleavage (perhaps by the acyl hydrolase domain of SolE) releases the β-keto-acid **4** and spontaneous decarboxylation would give the observed keto-amine **6** (Figure 4).

There remains one module of the PKS SolA and it is unclear what function this has. It is possible that it simply passes the acyl group on unchanged, which is quite common in *trans*-AT PKSs.^8,14^ SolA terminates with a domain of unknown function that is more often found in NRPSs than PKSs, which perhaps suggests that this end of SolA interacts with an NRPS such as SolF. The adenylation domain that starts SolF (Figure 7) has no clear predicted substrate.^49^ One possibility is that thioester **5** gets hydrolyzed to the corresponding acid, which is the carboxylic acid that is activated by the first adenylation domain of SolF. However, if this were correct, one would expect accumulation of the acid (hydrolyzed **5**) in the *solF* knockout strain. The fact that accumulation of aldehyde **2** derived from thioester **1** was detected suggests that the PKS was stalled due to its inability to pass on its final acyl group. Whether the acyl group gets passed directly to SolF or proceeds via the standalone ACP SolD is unknown. The next chain elongation clearly occurs on SolF (module 4, Figure 7) because this adenylation domain is predicted to activate glycine and because it contains the flavin monooxygenase (FMO) domain needed to produce the α-hydroxyglycine of solanimycin B.^49^ FMO domains are occasionally found in PKS modules, where their role is to hydroxylate α to the thioester. Examples of FMO domains in NRPS modules are found in the myxothiazol A and melithiazole A biosynthetic clusters. In both cases, the hydroxylation is on the α position of a glycyl residue, but in both cases, the hydroxylation is followed by cleavage of the carbinolamide to release the foregoing peptide as its C-terminal amide and leaving a two-carbon glyoxyl thioester which is hydrolyzed by the following thioesterase domain.^16,50^ In solanimycin B biosynthesis the cleavage of the carbinolamide does not occur and many further rounds of peptide- and polyketide-chain extension occur before the final product is released. In the myxothiazol A and melithiazole A biosynthetic clusters the FMO domain is inserted into a loop of the adenylation domain between the a4 and a5 motifs, whereas in SolF it is inserted between the a8 and a9 motifs. This is a more common position to find inserted domains such as methyl transferase domains, ketoreductase domains and oxidase domains.^50^ In the case of skyllamycin, which does retain the α-hydroxyglycine residue, there is no FMO domain in the NRPS but the gene cluster codes for a standalone FMO which has been shown to be responsible for this hydroxylation reaction.^36^

Solanimycin A is produced by skipping this FMO step, so it must be the case that the next condensation domain can accept either the hydroxylated or non-hydroxylated peptide. It is interesting that the ratio of solanimycin B to A was considerably lower when labeled serine (or glycine which can be converted to serine) was added to the medium. This suggests that without added serine the next NRPS module may be slow to load its serine residue, which means that the glycinamide group remains attached to module 4 for a longer period of time on average, giving more opportunity for the hydroxylation by the FMO domain to occur.

Following the (hydroxy)glycine residue are the five dehydroalanine residues. As previously mentioned, all dehydroalanine residues studied to date are derived by dehydration of a serine residue and the next NRPS module (SolG, module 5 in Figure 7) is predicted to insert serine. However, there is only one such module in the cluster (and there is only one other NRPS A-domain in the entire genome that is predicted to activate serine^49^), so it would seem that this module acts iteratively a total of five times. To provide evidence that the genome of *D. solani* MK10 has not been misassembled and that there are not multiple copies of module 5, we amplified the *solG* gene by PCR using primers matching the two ends of the gene. The DNA produced was of the expected size for a single module by gel electrophoresis and expression of the gene in *Escherichia coli* gave a predominantly monomeric protein of the expected size by SDS-PAGE, blue native PAGE, analytical ultracentrifugation and non-denaturing mass spectrometry (Figures S28–S33). So, there is no evidence of multiple copies of module 5.

There are NRPSs which are called “iterative” (or Type B)^15^ but in bacterial examples it is not single modules that iterate, but the completed peptide (made in the standard linear fashion) is held by the TE domain and coupled with the next peptide(s) to be synthesized, forming (generally cyclic) oligomers, from dimers up to pentamers. There are some cases, however, where iterative behavior of chain-extension modules is observed.^17^ For example, in the biosynthesis of the tri- and tetra-meric fungal depsipeptides beauvericin and bassianolide, there is no TE domain but instead a terminal C-domain, which transfers the peptide on module 2 backward, acylating the aminoacyl group attached to module 1.^51^ In the case of the locillomycins, a whole three-module protein (LocB) appears to act iteratively, adding D-Gln-L-Asp-Gly first time through and L-Asn-L-Asp-Gly second time through.^52^ Similarly in thalassospiramide biosynthesis there is a three-module section of TtcB that iterates once or twice.^53^ Finally in ε-poly-l-lysine biosynthesis the NRPS has domains A-T-C1-C2-C3 but the C-domains have low similarity to normal C-domains and the lysyl residue is transferred to an oligo-lysine chain that is free in solution and not attached to a carrier protein.^54^

The mechanism by which a single NRPS module incorporates more than one amino acid is unknown. In iterative PKSs the KS domain has a nucleophilic cysteine residue to which the acyl group is transferred (Figure 2a) and remains attached while the ACP is reloaded with a further chain-extending unit. However normal NRPS condensation domains do not have a nucleophilic residue and do not employ the same type of ping-pong mechanism. Instead, they catalyze direct acyl group transfer from the acyl donor to the acyl acceptor (Figure 2b). We suggest that the most likely mechanism is that two (or more) protomers of SolG dock onto each other and the peptidyl group attached to the first protomer gets passed onto the amino group of the serine attached to the second protomer, catalyzed by the condensation domain of the second protomer, and so on. This last mechanism is analogous to the mechanism of beauvericin and bassianolide biosynthesis and is what was proposed in the case of thalassospiramide as well. However, for iteration of two or more modules, the docking and transfer could be intramolecular within the same protein molecule, whereas it could not be intramolecular if a single module (with a single thiolation domain) is involved.

Following attachment of each serine residue, dehydration to dehydroalanine is required. This dehydration step is well characterized in the biosynthesis of RiPPs and occurs after activation of the hydroxyl group (by either phosphorylation or glutamylation) to make it a better leaving group.^25,55^ However there are no homologues of these enzymes in the solanimycin cluster. In NRPS products where such dehydration occurs during the biosynthesis, no separate enzyme or domain has been found to effect the dehydration and it has been assumed that one of the C domains catalyzes dehydration as well as condensation.^56,57^ In nocardicin biosynthesis, there is experimental evidence that the downstream C domain effects the dehydration (as well as, in that case, subsequent nucleophilic addition of the amino group of the downstream amino acid at the β-position of the dehydroalanine and finally amide bond formation to make a β-lactam).^24,58^ Because of this precedent, it is quite likely that the C domain of SolG is the domain that catalyzes dehydration. However, another possibility is that it is SolK, which is annotated as an enoyl hydratase but could equally well catalyze the reverse reaction, i.e., dehydration. In-frame deletion of *solK* resulted in approximately 73-fold lower production of solanimycins, showing it is important but not absolutely essential to the biosynthesis.

Phylogenetic analysis of the C domain of SolG on the NaPDoS server^56^ indicated that it is closest to the second C domain of the syringomycin NRPS, SyrE, in the “dual” clade. This clade of C domains catalyzes epimerization as well as amide bond formation, and epimerization, like dehydration, requires removal of the α-H. Therefore, the C-domain of SolG does appear to be a possible candidate for catalyzing dehydration. In fact, the C-domains of SolF, SolG, and the first C-domain of SolH are very closely related to each other (49–55% identity) and all fall in the dual clade. They all have HHxxDDx in place of the normal HHxxxDG catalytic motif. If dehydration is catalyzed by the downstream C-domain, then the fifth serine residue should be dehydrated by the first C-domain of SolH, which also seems possible, given its similarity to the C-domain of SolG.

Whichever protein it is that catalyzes the dehydration, it is likely that it occurs after the amide bond to the upstream amino acid has been formed but before the amide bond to the downstream amino acid. This is because dehydration while there is still a free amino group would give an enamine which would be prone to spontaneous hydrolysis, whereas when the thioester reacts to form an amide, the *pK*_a_ of the α-H rises and makes dehydration considerably more difficult.

The remainder of the biosynthesis cannot be deduced as the structure of the C-terminal group is unknown, except that it includes, and probably starts with the addition of valine by the one further module of NRPS (SolH). Thereafter, the remainder of the C-terminal group is probably a polyketide chain as substantial incorporation of [1,2-^13^C_2_]acetate was observed. It is not likely that the polyketide portion is attached to the peptide via an amide or ester linkage to the valine because otherwise fragmentation at this amide/ester should have been observed in the MS–MS. Therefore, polyketide chain extension of the peptidyl thioester is probable. SolA is the only PKS in the cluster, but the first two modules have been proposed as the source of the N-terminal polyketide. Therefore, it is possible that module 3 is an iterative PKS that completes the biosynthesis, but it would be unusual for the acyl starter unit to be passed to a PKS module in the middle of a larger PKS—this would normally occur at the N-terminal module of a PKS. Also, to get the required formula, some additional reductase, in addition to the KR in module 3, would be needed, either an enoyl reductase or a thioester reductase. Neither type of reductase is found in the cluster. Maybe a reductase from outside the cluster is involved or perhaps some PKS that is not in the cluster is responsible for the polyketide chain extension. However, it should be noted that the transposon mutagenesis that identified the cluster did not identify any other genes outside the cluster that were essential for the biosynthesis.

Further work on the solanimycins should focus on (a) identification of the C-terminal fragment, (b) proof that the proteins encoded by the genes in the cluster do catalyze the proposed reactions, and (c) what other gene product(s) are involved in the biosynthesis. Preliminary studies toward (a) revealed that hydrolysis of semi-purified solanimycin in 2 M hydrochloric acid led to a new peak in LC–MS with the mass expected for the C-terminal amine (e.g., **10**), which was not observed for the *sol^–^* strain (Figure S34). These studies need to be repeated on a larger scale to allow sufficient quantity of this amine to be purified for structure determination. Toward (b), we have showed that SolG can be expressed in *E. coli* in soluble form and is largely monomeric but the investigation of its catalytic activity is still needed. The authors are happy to share data with the research community, on request, so that studies on this topic can continue.

In summary, we have identified much of the structure of solanimycins A and B from LC/MS/MS and identification of shunt metabolites produced by gene-deletion strains. This revealed that the cluster responsible for generating the solanimycins is a noncanonical PKS-NRPS system and the product structures deviate substantially from those that were proposed^13^ based on bioinformatic analysis. Most remarkably, the central portion is a hexapeptide containing five sequential dehydro-α-alanine residues. Isotopic labeling showed that these are derived from serine as expected. As the gene cluster contains genes coding for NRPS modules and no gene coding for the precursor protein of a RiPP, it seems that the peptide portion must be NRPS-derived. Only one NRPS module in the cluster is predicted to activate and incorporate serine (SolG), so we conclude that this module must act iteratively, five times in a row. This is, we believe, unprecedented and would represent a change in our understanding of what an NRPS module can do.

## Supporting Information Available

Methods for production, purification, and anti-fungal assay of solanimycin and shunt metabolites; MS and MS/MS, NMR, and UV spectra; and isotopic labeling experiments and protein expression and analysis (PDF)

## Abbreviations

PKS: polyketide synthase
ACP: acyl carrier protein
NRPS: nonribosomal peptide synthetase
AT: acyl transferase
Dha: dehydroalanine
RiPP: ribosomally derived and post-translationally modified peptide. See caption to Figure 1 for further domain abbreviations.
