# Supplementary material for: Biosynthesis of Antifungal Solanimycin May Involve an Iterative Nonribosomal Peptide Synthetase Module
Source: ACS Chem Biol. 2023 Apr 17;18(5):1148–57. doi: 10.1021/acschembio.2c00947 (PMC10204066; doi:10.1021/acschembio.2c00947)
Supplement: Supplementary file 1 — cb2c00947_si_001.pdf [file cb2c00947_si_001.pdf]

Supporting Information

Biosynthesis of Antifungal Solanimycin May Involve an Iterative Non-Ribosomal Peptide Synthetase (NRPS) Module

Annabel C. Murphy[#+¶], Matthew Corney[#], Rita E. Monson[§], Miguel A. Matilla[§†], George P. C. Salmond[§] and Finian J. Leeper[#*]

[#] Yusuf Hamied Department of Chemistry, University of Cambridge, Lensfield Road, Cambridge CB2 1EW, U.K.

[§] Department of Biochemistry, University of Cambridge, Tennis Court Road, Cambridge CB2 1QW, U.K.

* Corresponding author, e-mail: fjl1@cam.ac.uk

Contents

Bacterial and Fungal Strains used in this study (Table S1) S2
General methods and assay for anti-fungal activity S2
Optimised solanimycin production and partial purification S3
MS data for solanimycin (Figs. S1-3) S3
Isolation and identification of shunt metabolites from mutant strains (Figs. S4-12) S5
UV and NMR data for solanimycin (Figs. S13-16) S9
Synthesis of [U-^{2}H]glycine S12
Feeding of [3,3-$^2H_2$]-L-serine (Figs. S17-18 & Table S2) S13
Feeding of [$^2H_2$]glycine and [$^{13}C_2$]glycine (Figs. S19-23 & Table S3) S14
Feeding of d_8-L-valine (Figs. S24-25) S17
Feeding of sodium [$^{13}C_2$]acetate (Figs. S26-27 & Table S4) S19
Expression and Analysis of SolG (Figs. S28-33) S20
Hydrolysis of solanimycin (Fig. S34) S25
References S26

Table S1. Bacteria and fungi used or referred to in this study

Strain	Here called	Genotype or relevant characteristic[a]	Reference or source
Saccharomyces cerevisiae		Wild type	S. Oliver
Dickeya solani MK10		Wild type, plant pathogen	(1)
MK10-OocN		MK10 transposon mutant *oocN*::Tn-KRCPN1; oocydin A negative, Km^R	(2)
OocN-M3	*ooc*⁻/*sol*⁻	MK10 transposon mutant *oocN*::Tn-KRCPN1, *solA*::mini-Tn*5*Sm/Sp; Km^R, Sm^R	(3)
OocN-Δ*solB*	*ooc*⁻/*solB*⁻	MK10 in-frame Δ*solB* (828 bp Δ), *oocN*::Tn-KRCPN1; Km^R	(3)
OocN-VfmG		*D. solani* MK10 *lacZ*::mini-Tn*5*, *vfmG*::Tn-DS1028; *oocN*::Tn-KRCPN1; Km^R, Sm^R, Cm^R	(3)
OocN-VfmG-Δ*solF*	*vfmG*/*solF*⁻	MK10 in-frame Δ*solF* (1701 bp Δ), *lacZ*::mini-Tn*5*, *vfmG*::Tn-DS1028; *oocN*::Tn-KRCPN1; Km^R, Sm^R, Cm^R	(3)

[a]Cm, Chloramphenicol; Km, kanamycin; Sm, streptomycin; Sp, spectinomycin.

1. Pritchard L.; Humphris S.; Baeyen S.; Maes M.; Van Vaerenbergh J.; Elphinstone J.; Saddler G.; Toth I., Draft Genome Sequences of Four *Dickeya dianthicola* and Four *Dickeya solani* Strains. *Genome Announc*., **2013**, *1*, e00087-12.
2. Matilla M. A.; Fang X.; Salmond G. P., Viunalikeviruses are environmentally common agents of horizontal gene transfer in pathogens and biocontrol bacteria. *ISME J*., **2014**, *8*, 2143–2147.
3. Matilla, M. A.; Monson, R. E.; Murphy, A. Schicketanz, M.; Rawlinson, A. Duncan, C.; Mata, J.; Leeper, F. J.; Salmond, G. P. C., Solanimycin: biosynthesis and distribution of a new antifungal antibiotic regulated by two quorum sensing systems, *mBio*, **2022**, e02472-22. DOI 10.1128/mbio.02472-22

General Methods

High resolution mass spectrometry was carried out using a Waters Acquity UPLC system using a BEH C18 1.7 µm 2.1 x 50 mm column coupled to a Waters Xevo G2-S QTOF MS running in positive mode with an electrospray source. UPLC separation was carried out with an 8 min program consisting of a 10 to 90% gradient of MeCN over 7.2 mins, 100% MeCN to 7.6 mins and finally re-equilibration to 10 % MeCN. The MS source had a temperature of 120 °C, capillary voltage of 3 kV and sampling cone voltage of 40 V. Fragmentation MS/MS experiments (CID) were run with a collision energy ramp of 15-40 V and fixed masses for detection were selected as appropriate.

NMR experiments were carried out on a Bruker Avance 500 MHz TCI Cryoprobe, except for experiments where high sensitivity for ^{13}C was required (i.e. ^{13}C COSY), in which case a Bruker 500 MHz DCH Cryoprobe Spectrometer was used.

HPLC purification was carried out on an Agilent 1100 series with a Phenomenex Jupiter 5 µm 4.6 x 250 mm C18 column.

UV spectra were obtained on a Cary 100 Bio UV-vis spectrometer.

Assay for anti-fungal activity

Standard yeast extract agar (25 mL, 5 g/L yeast extract, 20 g/L glucose) was overlaid with yeast extract agar (5 mL) containing 0.8% agar and 100 µL of an overnight seed culture of *Saccharomyces cerevisiae* grown in yeast extract (5 mL). Once set, wells were cut using a cork borer and 100 µL of the sample of interest placed inside the well. The plate was then incubated at 30 °C for 24 h. Samples to be tested were routinely made up in 20% ethanol solution.

Optimised solanimycin production and partial purification

Seed cultures of *Dickeya solani* MK10 *ooc*$^{-}$ were prepared by inoculation of 5 mL LB in 30 mL tubes and incubation in a rotary shaker overnight at 30 °C. Seeds were used to inoculate (1%) 100 mL media containing 5 g/L potato dextrose supplemented with 10% of standard LB broth (prepared and autoclaved separately) in 500 mL unbaffled conical flasks covered with tin foil. Cultures were shaken at 22 °C and 215 rpm for 24 h. After centrifugation, the supernatant was clarified using sterile filtration, then lyophilised. The resulting residue was extracted with methanol (approx. 250 mL per L supernatant), then after filtration to remove the residue, the solvent was removed by rotary evaporation to provide a yellow/orange thick oil (1.5 g/L supernatant). After dilution in a minimal volume of methanol, the crude extract was purified on an LH20 column (40 g resin/g extract) using methanol as eluent. Active fractions were identified using cut-well plate assays and combined. After removal of methanol *in vacuo* a yellow oil was obtained (approximately 0.7 g/L supernatant). The extract was then purified on a C18 resin pad (25 g/L supernatant) using a stepwise gradient between 20% and 100% acetonitrile (activity observed for fractions between 70 and 100% acetonitrile). After removal of solvent by rotary evaporation a yellowish oil was obtained (5-10 mg/L supernatant). NMR data and mass spec data was obtained at this stage for both MK10 *ooc*$^{-}$ and, following an identical protocol, for MK10 *ooc*$^{-}$/*sol*$^{-}$. The material used for these studies derived from processing of a total of 3 L of culture for each mutant.

MS data for solanimycin

Comparison of LCMS traces for extracts of MK10 *ooc*$^{-}$ and MK10 *ooc*$^{-}$/*sol*$^{-}$ cultures purified using LH20 and C_{18} resin allowed two ions to be identified that were only present in the *ooc*$^{-}$ extracts. These had *m/z* of 963.48 and 979.474 and extracted ion chromatograms are shown in **Fig. S1** and **S2**. The difference in molecular weight of these ions corresponds accurately to the difference of one oxygen atom.

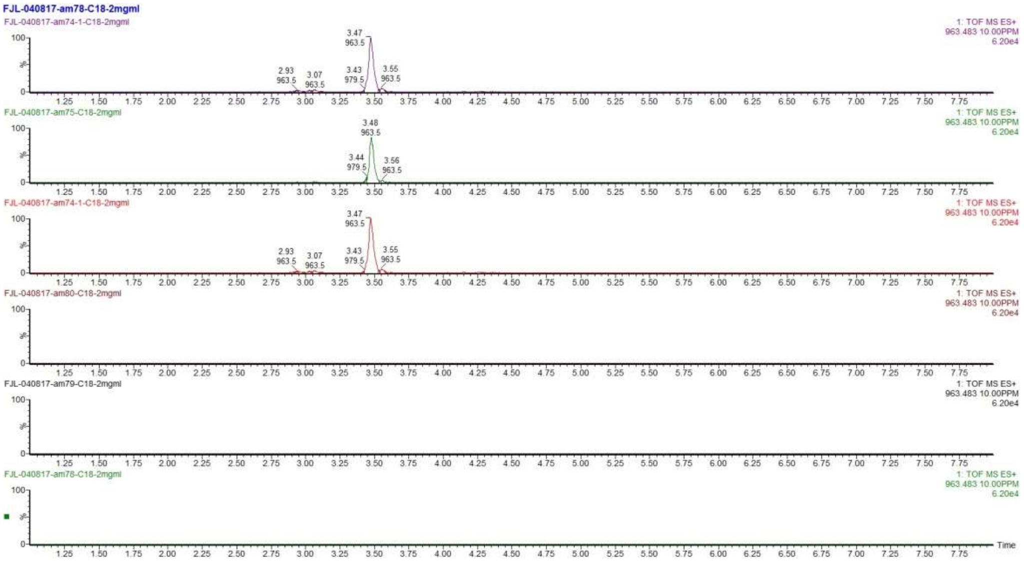

Fig. S1. Extracted ion chromatograph for ions at 963.48 observed for triplicate purified extracts of cultures of *D. solani* MK10 *ooc*$^{-}$ (top 3 traces) and *D. solani* MK10 *ooc*$^{-}$/*sol*$^{-}$ (bottom 3 traces).

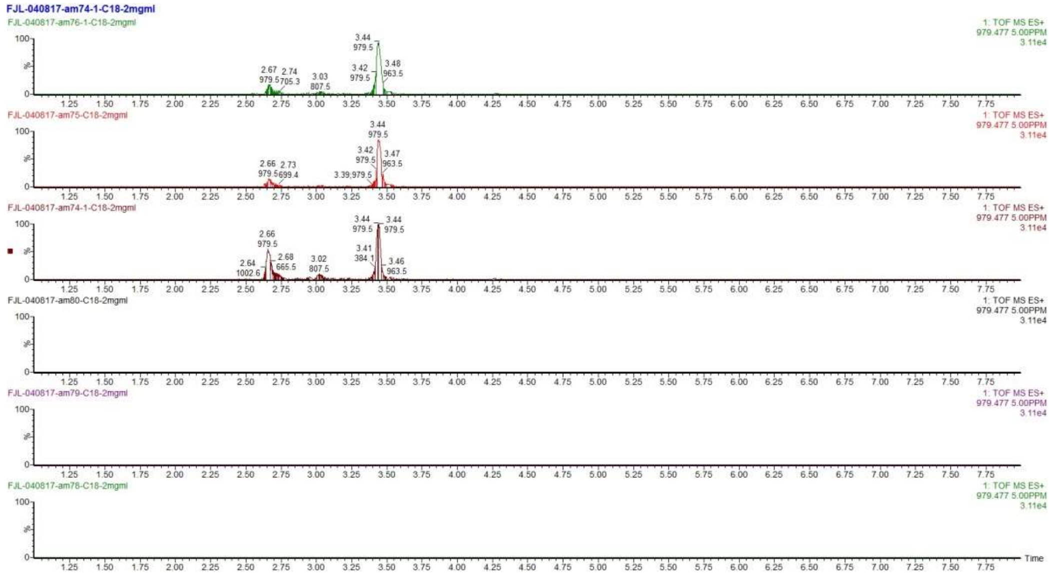

Fig. S2. Extracted ion chromatograph for ions at 979.477 observed for triplicate purified extracts of cultures of MK10 *ooc*⁻ (top 3 traces) and MK10 *ooc*⁻/*sol*⁻ (bottom 3 traces).

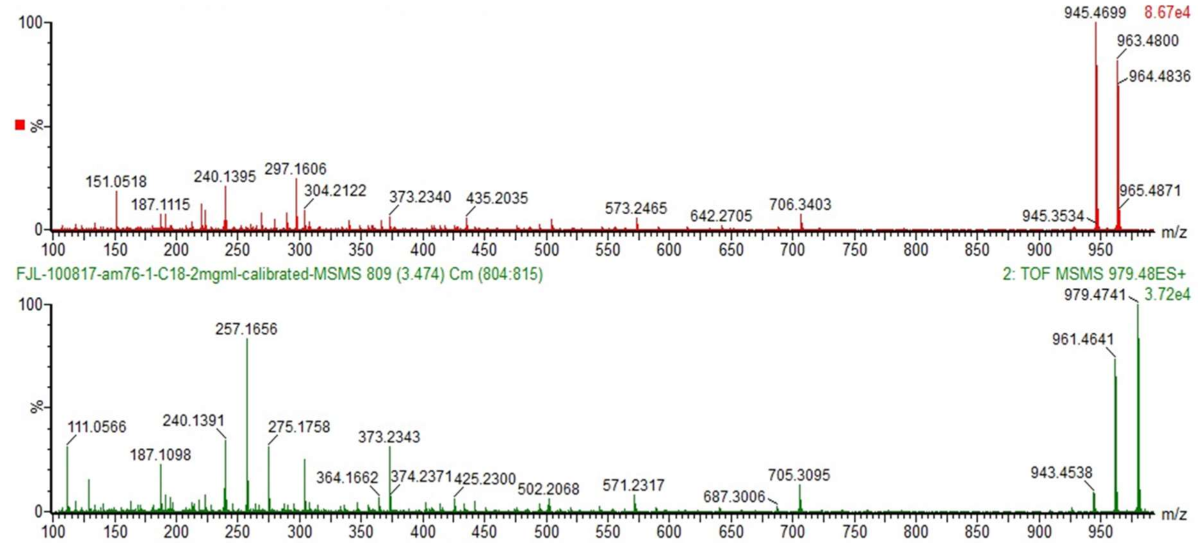

Figure S3. Collision induced dissociative fragmentation of: (top) solanimycin A (*m/z* 963.48) and (bottom) solanimycin B (*m/z* 979.474) partially purified from cultures of MK10 *ooc*⁻.

Shunt metabolite isolation from solB and vfmG/solF mutants

Tetradec-2,4,6,8,10,12-hexaenal

D. solani MK10 *vfmG/solF*⁻ (*vfmG* is a regulator gene and strains where it is mutated produce higher levels of solanimycin and of shunt metabolites) was fermented in a total of 1.8 L media as described in the general methods above with the exception that the media contained 14 g/L potato dextrose in place of 5 g/L. After centrifugation, the yellowish cell pellet was lysed and incubated for 30 mins with ethanol containing 4% 1 M HCl. The cell debris was removed by centrifugation and the resulting bright orange solution was evaporated *in vacuo*. The extract was taken up in water (acidic due to residual HCl) and extracted 3 times with ethyl acetate, then the aqueous layer basified using 1 M NaOH and extracted again. The combined organic extracts were dried ($MgSO_4$) and evaporated *in vacuo* to give a yellow extract (24 mg). This was purified on silica using 5-20% ethyl acetate in hexane, to give 1.5 mg partially purified material. This was then purified by HPLC using a gradient of MeCN and water containing 0.1% formic acid as follows: 5 to 98% MeCN over 6 mins, followed by 98% MeCN for 6 mins. The yellow pigment was collected as two closely eluting fractions between 10 and 11 mins. Only the second fraction had sufficient material for characterization by ^{1}H, COSY and HSQC NMR and NMR showed it remained impure. However, the structure could be elucidated based on this data.

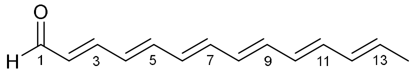

Yield ≤ 1 mg, R_f 0.75 (silica, 4:1 hexane:ethyl acetate); δ_H ppm (500 MHz, $CDCl_3$) 9.56 (1H, d, 8.0 Hz, H1), 7.14 (1H, dd, 11.3, 15.0 Hz, H3), 6.72 (1H, dd, 11.3, 15.0 Hz, H5), 6.53 (1H, dd, 11.2, 14.6 Hz), 6.50-6.10 (8H, m), 5.82 (1H, dq, 7.3, 14.6 Hz, H13), 1.81 (3H, d, 7.2 Hz, H14); δ_C ppm (125 MHz, $CDCl_3$) 193 (CHO), 151.9 (CH, C3), 142.9 (CH, C5), 139.1 (CH), 137.0 (CH), 135.7 (CH), 132.1 (CH, C13), 131.8 (CH), 131.5 (CH), 131.0 (CH), 130.7 (CH, C2), 130.1 (CH), 129.5 (CH, C4), 18.5 (CH_3, C14); λ_{max} 390 nm; LR-ESI-MS *m/z* = 201.2 $[M+H]^+$ (calc. for $C_{14}H_{17}O$ 201.1279); HR-ASAP-MS *m/z* = 200.1200 $[M]^-$ (calc. for $C_{14}H_{16}O$ 200.1201).
Note: the coupling constants that were measurable were consistent with the all-*trans* geometry shown but not all coupling constants could be measured due to peak overlap.

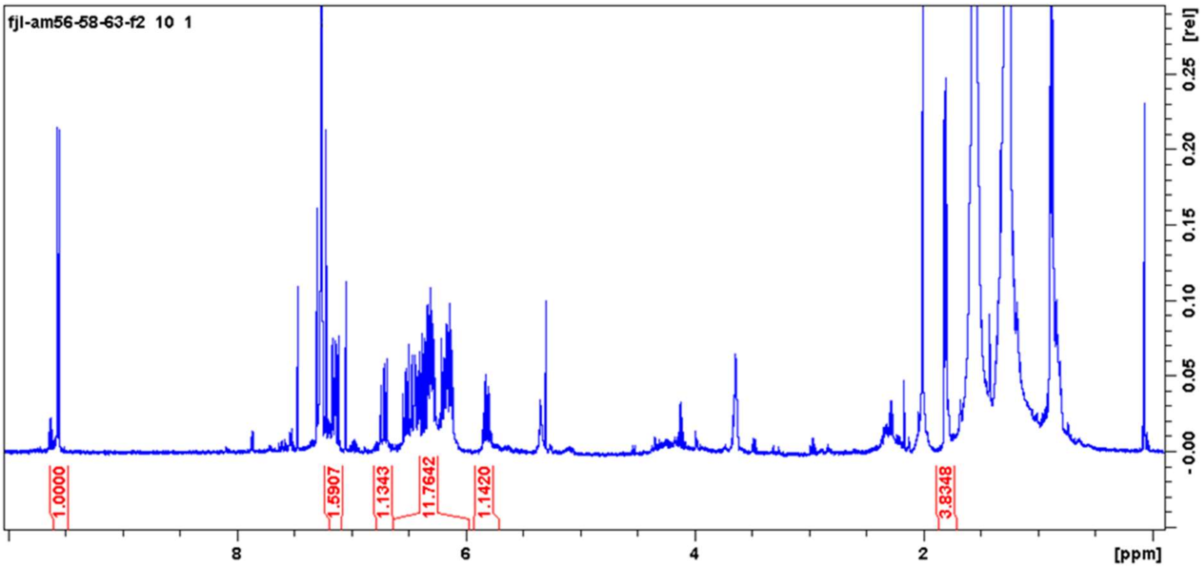

Figure S4. ^{1}H NMR data for pigment isolated from MK10 *vfmG*/*solF*$^{-}$.

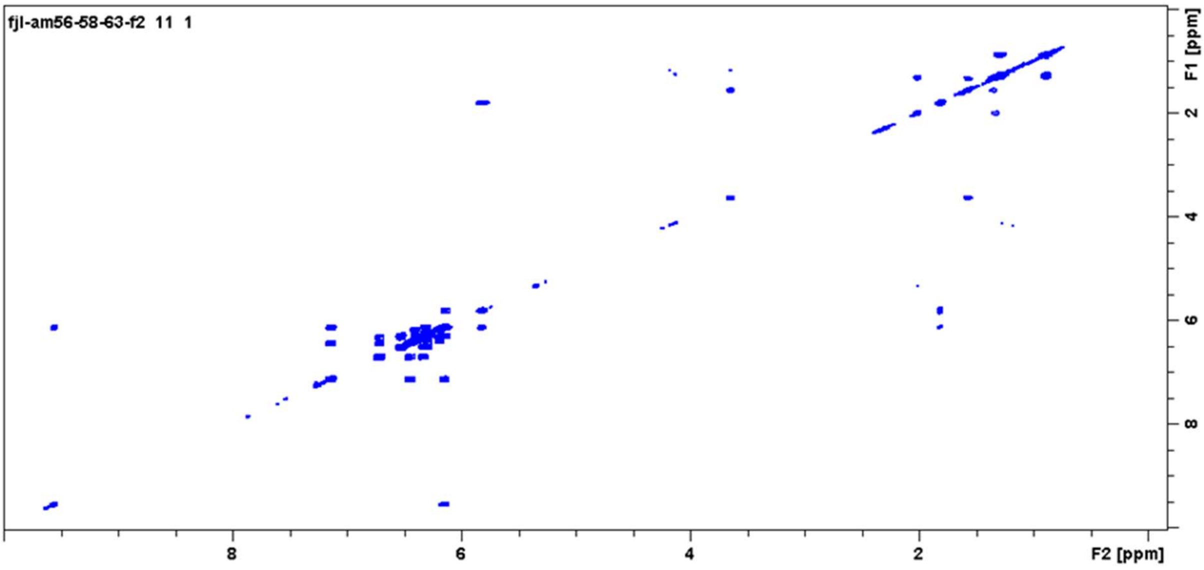

Figure S5. COSY NMR data for pigment isolated from MK10 *vfmG*/*solF*$^{-}$.

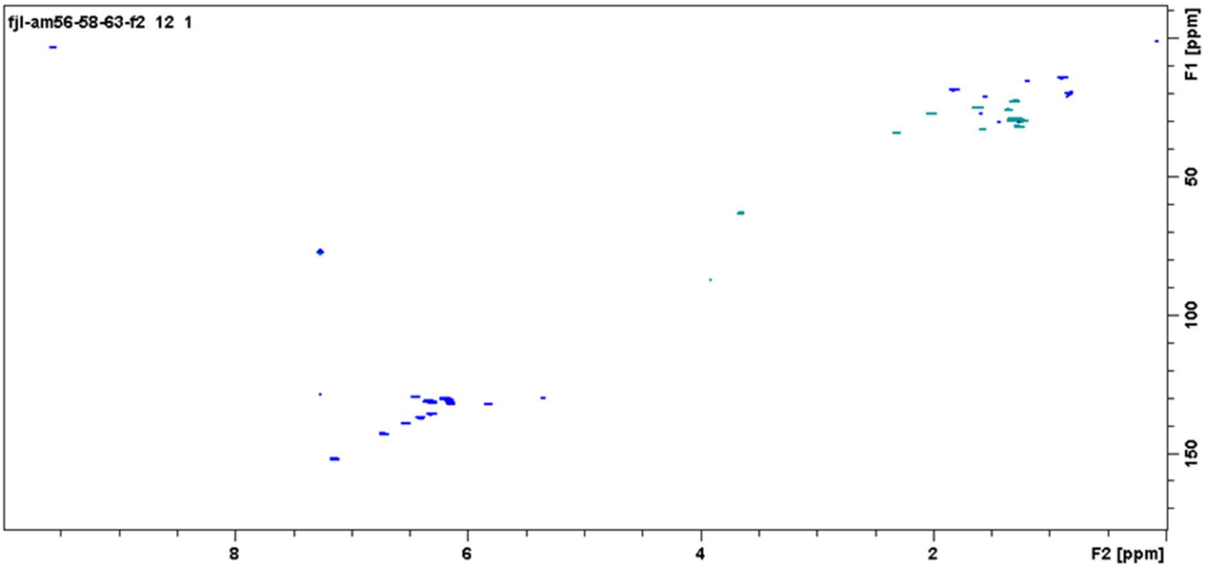

Figure S6. HSQC NMR data for pigment isolated from MK10 *vfmG*/*solF*$^{-}$.

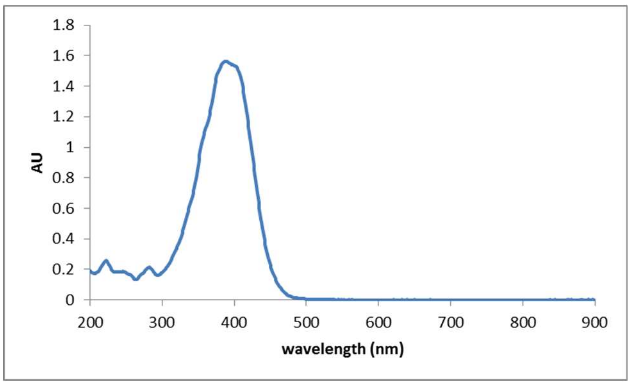

Figure S7. UV spectrum of the pigment isolated from MK10 *vfmG*/*solF*$^{-}$.

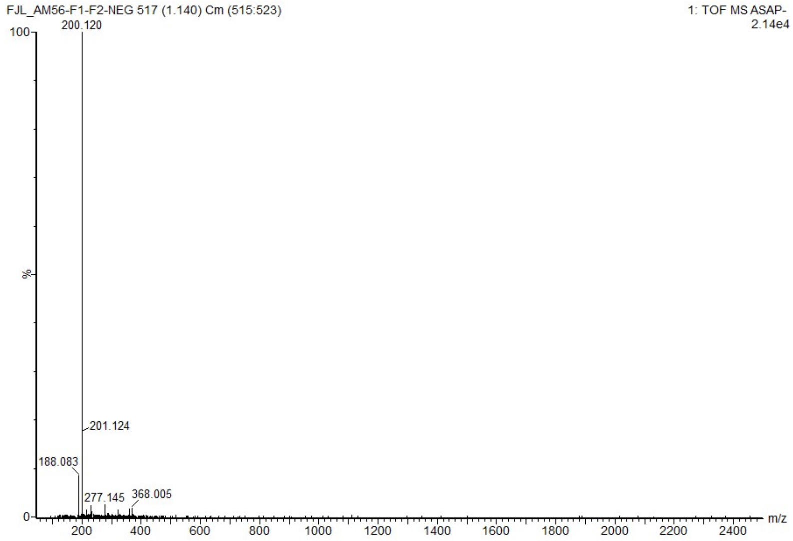

Figure S8. HRMS data for pigment isolated from MK10 *vfmG*/*solF*$^{-}$ using ASAP ionisation in negative-ion mode ([M]$^{-}$ observed).

Analysis of *D. solani* MK10 *ooc-/solB-* mutant

Culture, extraction and purification methods for *D. solani* MK10 mutants were as described for solanimycin production in the general methods.

Triplicate samples for the *solB*⁻ mutant and the *sol*⁻ mutant were analysed by low-res LCMS with UV detection (single replicate, **Fig. S9**) and high-res LCMS without UV detection. UV detection allowed the pigment to be identified as a pair of peaks which had an *m/z* of 230.2 (positive mode). High-resolution MS confirmed that two peaks at *m/z* 230.154 were present in all *solB*⁻ samples and none of the *sol*⁻ samples (**Figure S10**).

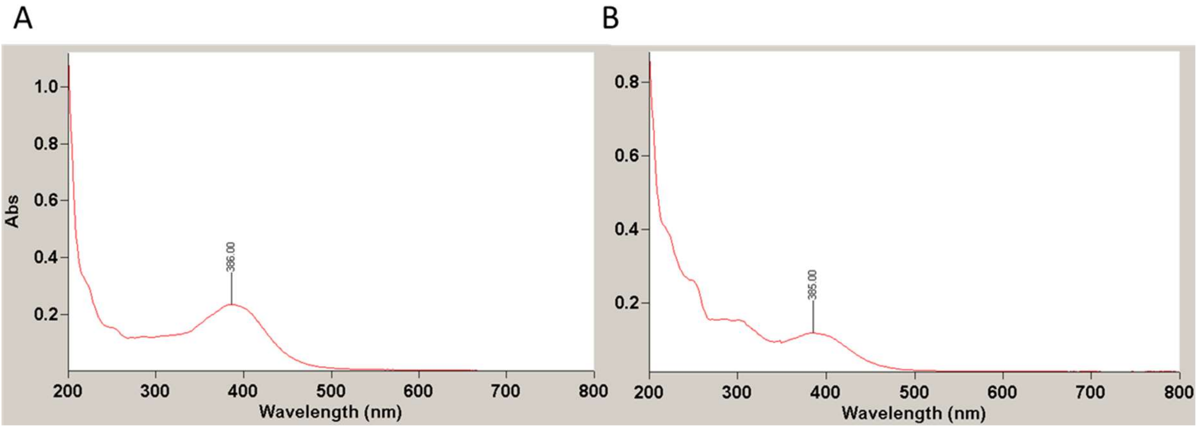

Figure S9. UV traces for two pigments (A, fraction 1; B, fraction 2) purified by HPLC from MK10 *ooc*⁻ */solB*⁻ mutant.

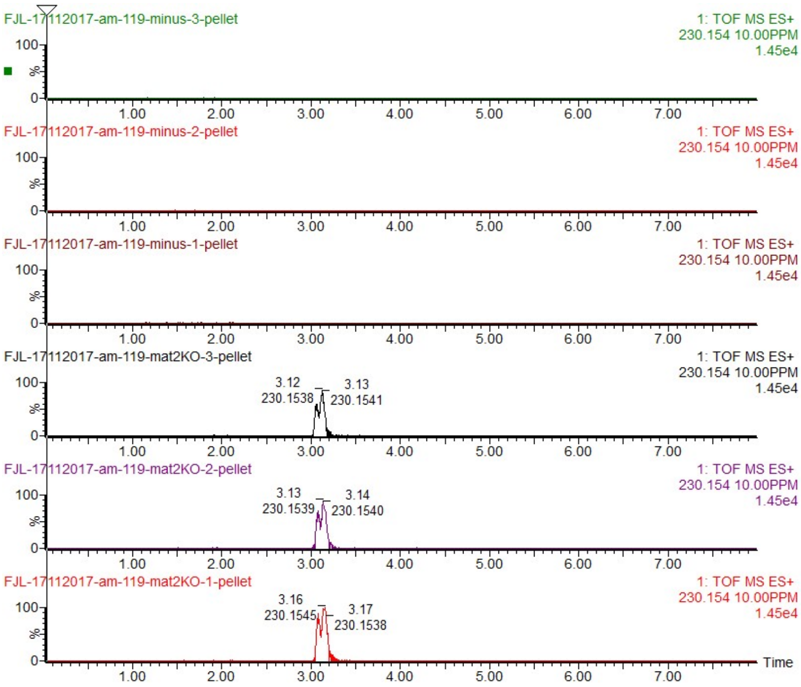

Figure S10. Extracted ion chromatogram of ion at *m/z* 230.154 for triplicate *D. solani* MK10 *ooc*⁻*/sol*⁻ (top three traces) and *solB*⁻ (bottom three traces) cell pellet extracts.

1-Aminopentadec-3,5,7,9,11,13-hexaen-2-one

Dickeya solani MK10 *ooc*$^-$/*solB*$^-$ was fermented in a total of 1L media as described in the general methods. After centrifugation, the yellowish cell pellet was lysed and incubated for 30 mins with ethanol containing 4% 1 M HCl. The cell debris was removed by centrifugation and the resulting bright orange solution was evaporated *in vacuo*. The extract was applied to silica equilibrated in ethyl acetate, washed with ethyl acetate and the yellow pigment eluted using 10% MeOH in ethyl acetate (R_f 0.48 CH_2Cl_2:MeOH, 9:1). This was then purified by HPLC using a gradient of MeCN/water containing 0.1% formic acid, as follows: 20 to 65.5% MeCN over 14 min, then 95% MeCN for 4 min. The yellow pigment was collected as two closely eluting fractions between 12 and 14 min.

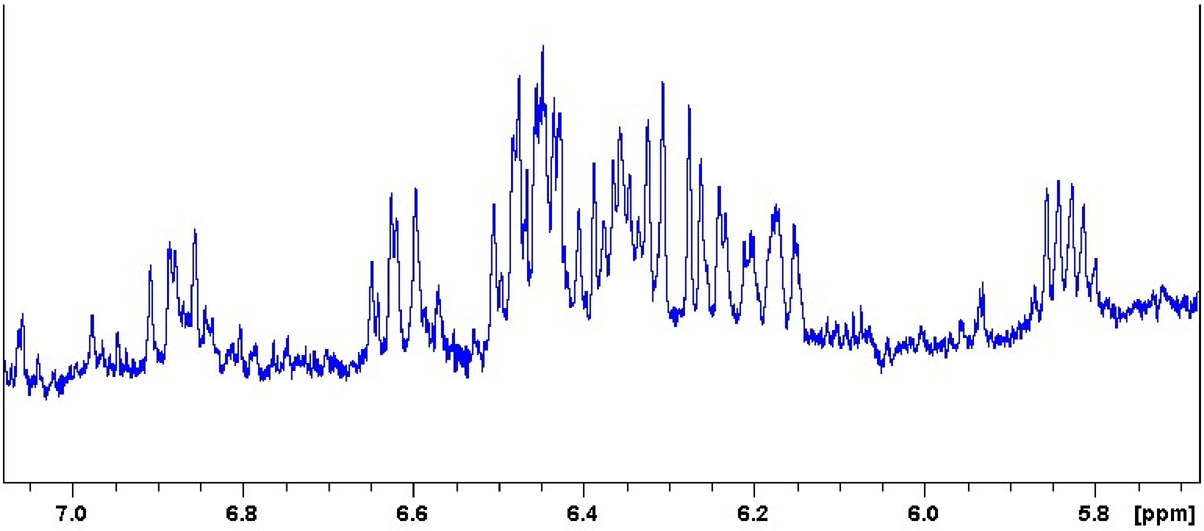

Figure S11. Partial ^{1}H NMR spectrum for one pigment (fraction 2) partially purified by HPLC from MK10 *ooc*$^-$/*solB*$^-$ mutant.

UV data for solanimycin

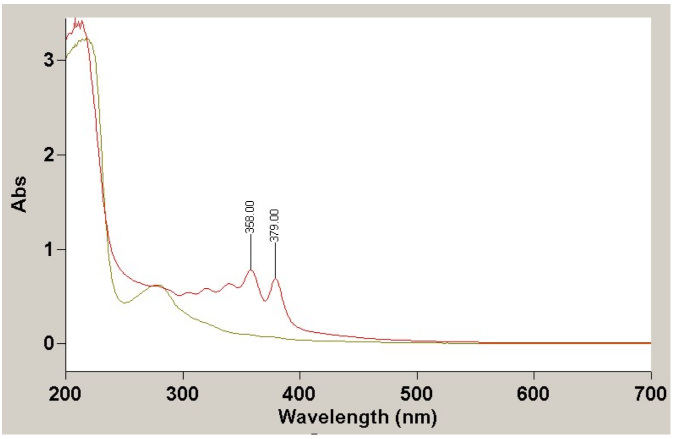

Figure S12. UV chromophore for partially purified extracts from MK10 *ooc*$^-$/*sol*$^+$ (red) and *ooc*$^-$/*sol*$^-$ (green) cultures, dissolved in 50:50 H_2O:MeCN.

NMR data for solanimycin

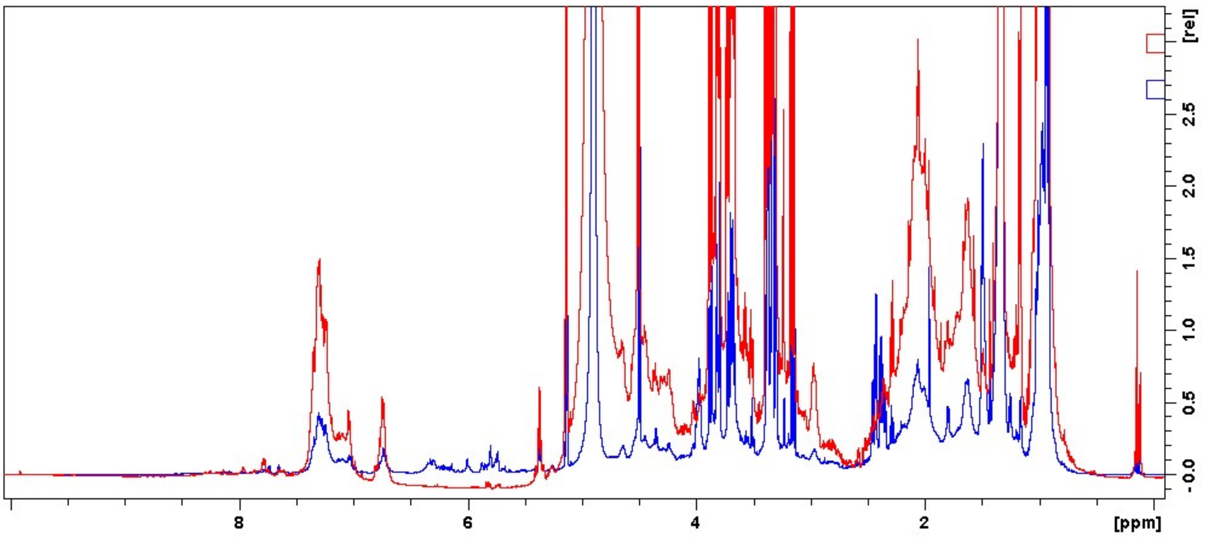

Figure S13. Overlaid ^{1}H NMR spectra of MK10 *ooc*$^-$/*sol*$^+$ (blue) and *ooc*$^-$/*sol*$^-$ (red) partially purified extracts in CD_3OD. Integration of this spectrum gave an integral for the 5.6-6.5 ppm region (the dehydroalanine and polyene protons of solanimycin) of 1.76 and an integral for all other non-solvent peaks of 82.4. The remaining protons of the solanimycins would have contributed an integral of about 2.4 to the other peaks, so the total integral due to the solanimycins would be 4.16 and the integral due to other compounds would be 80. Thus the mixture comprises about 5% solanimycins.

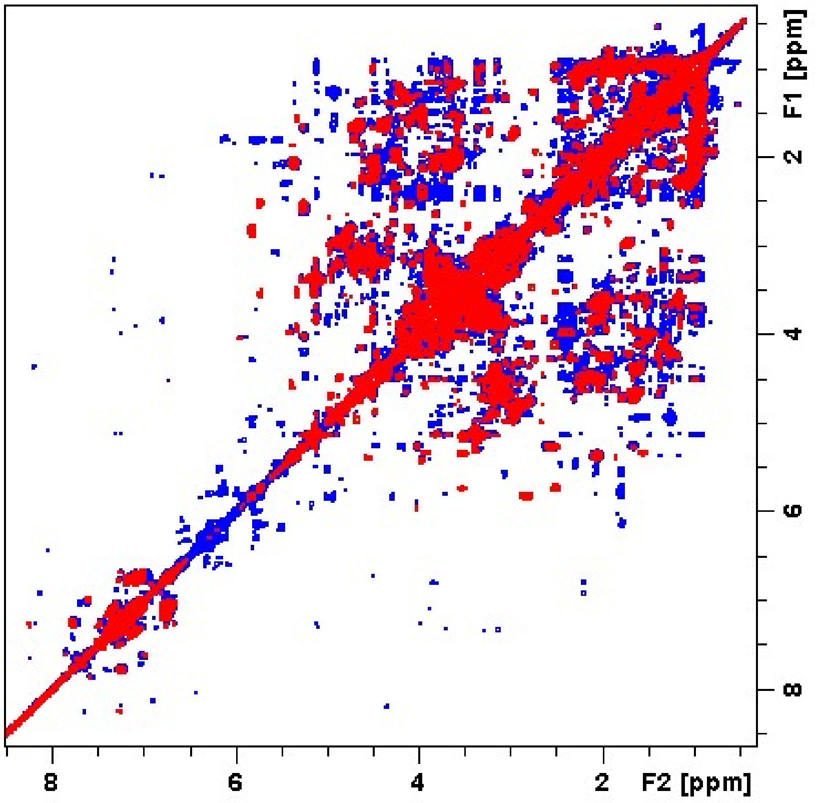

Figure S14. Overlaid COSY NMR spectra of MK10 *ooc*$^-$ /*sol*$^+$ (blue) and *ooc*$^-$/*sol*$^-$ (red) partially purified extracts in CD_3OD.

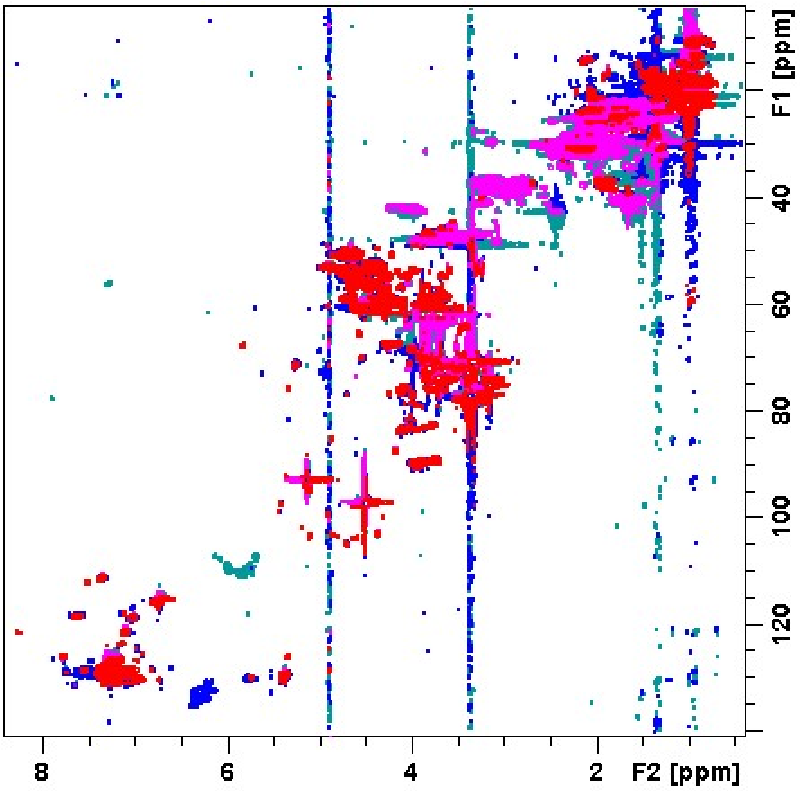

Figure S15. Overlaid HSQC NMR spectra of MK10 *ooc*$^{-}$ /*sol*$^{+}$ (blue/green) and *ooc*$^{-}$/*sol*$^{-}$ (red/pink) partially purified extracts in CD_3OD.

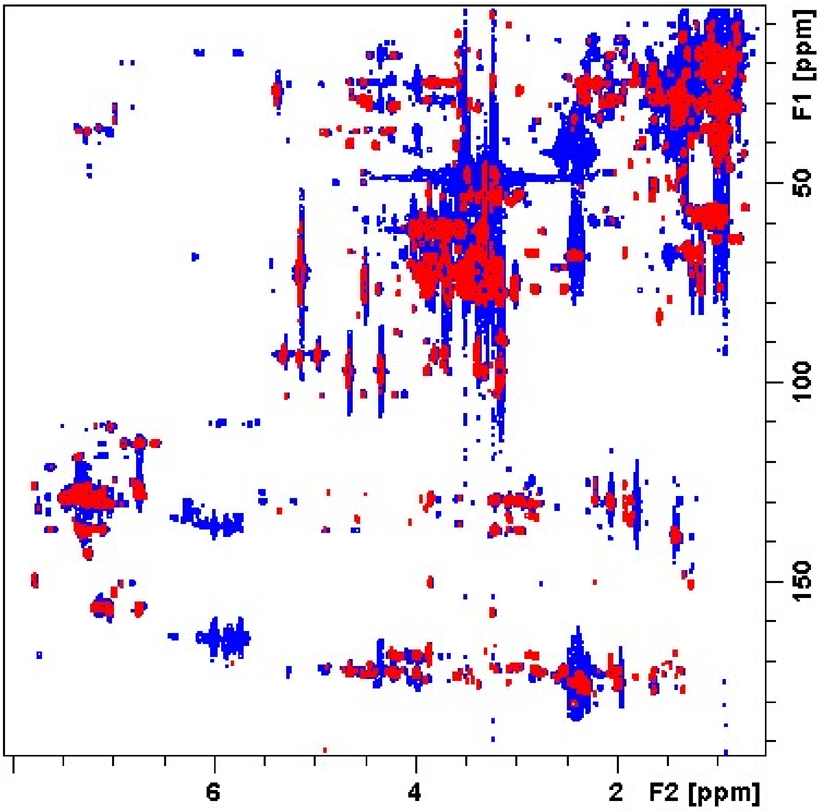

Figure S16. Overlaid HMBC NMR spectra of MK10 *ooc*$^{-}$ /*sol*$^{+}$ (blue) and *ooc*$^{-}$/*sol*$^{-}$ (red) partially purified extracts in CD_3OD.

A summary of the chemical shifts observed for the dehydroalanines, unhydroxylated glycine and partial data for the polyene motif are given in the table below. The dehydroalanines cannot be placed in order as no correlations between residues could be confidently assigned. Due to overlapping signals in the proton NMR, no coupling constants are provided. No signals could be confidently assigned to the hydroxylated glycine residue.

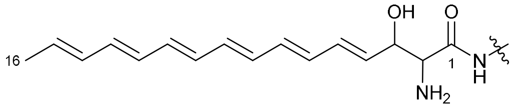

	δ_H (ppm)	δ_C (ppm)
**Dha_1**	6.03, 5.67 (H_β)	163.7 (C=O), 135.3(C_α), 107.6 (C_β)
**Dha_2**	5.97, 5.73 (H_β)	163.9 (C=O), 135.8 (C_α), 108.8 (C_β)
**Dha_3**	5.98, 5.79 (H_β)	164.3 (C=O), 135.8 (C_α), 109.5 (C_β)
**Dha_4**	5.81, 5.72 (H_β)	163.9 (C=O), 136.4 (C_α), 110.0 (C_β)
**Dha_5**	5.84, 5.78 (H_β)	165.1 (C=O), 135.6 (C_α), 110.3 (C_β)
**Gly**	4.05 (H_α)	175.5 (C=O), 42.5 (C_α)
**Polyene C6-C13**	6.15-6.41	130-136 (CH)
**Polyene C14**	6.13	132.0 (CH)
**Polyene C15**	5.75	133.5 (CH)
**Polyene C16**	1.78	17.2 (CH_3)

Stable isotope feeding studies

Synthesis of [U-^{2}H]glycine

Following the procedure given by Blomquist *et al., J. Org. Chem.*, **1966**, *31* (1), 338-339, diethyl acetamidomalonate (382 mg) was dissolved in D_2O (2 mL) and cooled on ice. Thionyl chloride (530 µL) was added dropwise and the reaction left on ice for 5 min. The ice was then removed and the reaction heated at reflux overnight. After cooling, most of the solvent was removed *in vacuo* and THF (7 mL) added. The mixture was cooled to 4 °C for 1 h, then the precipitated glycine was filtered and washed with diethyl ether. The solid material was dissolved in D_2O (200 µL) and triethylamine (270 µL), and the mixture was stirred at room temperature for 10 min. Acetone (2.5 mL) was added and the mixture was stirred for a further 10 min. The precipitate was filtered, washed with acetone, chloroform repeatedly, and finally ether. The resulting solid was dried in a desiccator overnight to give a white solid (98 mg). ^{13}C NMR showed that the correct product had been formed, with splitting of the alpha carbon into a quintet observed due to ^{2}H-^{13}C coupling.

Feeding studies were carried out by adding labelled material after cultures of *D. solani* MK10 *ooc*$^-$ had been allowed to grow for around 7 h, shortly before the onset of solanimycin production. The cultures were then processed and purified (LH20/C_{18} resin) as described above and analysed by HR-LCMS.

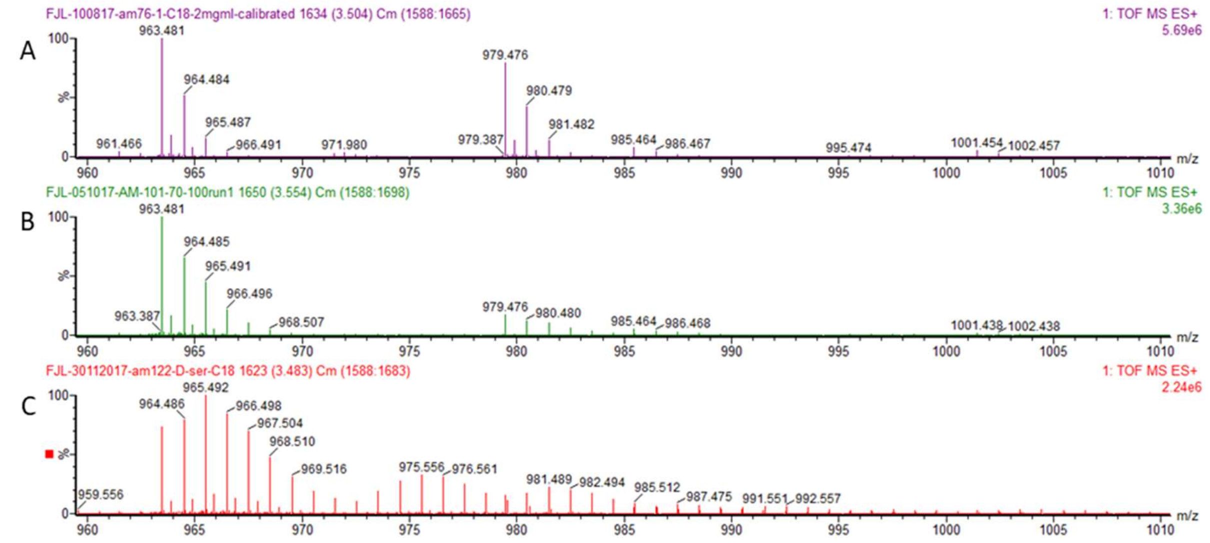

Figure S17. Observed isotope patterns for the 963 ion and the 979 ion for solanimycin isolated from MK10 *ooc*⁻ cultures that were (**A**) not fed any labelled substrate, or fed [3,3-$^2H_2$]-L-serine at (**B**) 2 mM or (**C**) 8 mM.

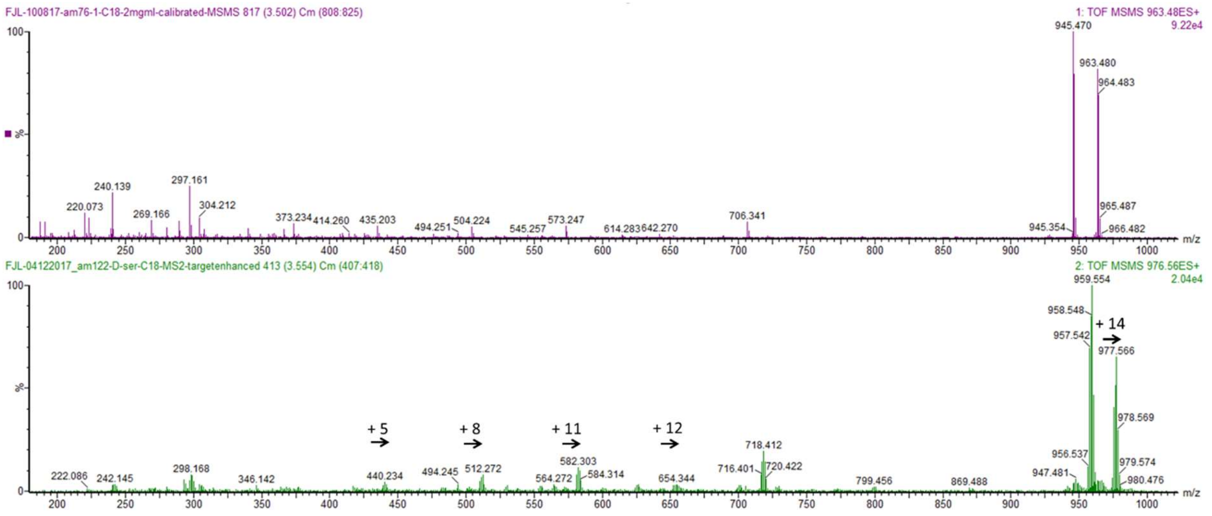

Figure S18. Fragmentation patterns for the 963 ion of solanimycin (top) compared with the fragmentation pattern (bottom) observed for a solanimycin ion (around A+14) that has incorporated deuterium as a result of feeding [3,3-$^2H_2$]-L-serine. Approximate increases observed for the labelled envelope are indicated relative to the natural abundance solanimycin ion.

Table S2. Abundances of ions related to the solanimycin, including ions containing natural abundance ^{13}C and ^{18}O and ^{2}H as a result of incorporation of [3,3-$^{2}H_2$]-L-serine.

	3,3-$^{2}H_2$-Ser				3,3-$^{2}H_2$-Ser		
963.480 (M)	0 mM	2 mM	8 mM	979.477 (M)	0 mM	2 mM	8 mM
M	100	100	73%	M	100	100	73%
M+1	52%	64%	79%	M+1	53%	71%	77%
M+2	15.5%	44%	100%	M+2	16.5%	53%	100%
M+3	3.6%	20%	84%	M+3	3.9%	27%	86%
M+4	0.7%	9%	70%	M+4	0.9%	13%	77%
M+5		3.5%	47%	M+5		6%	55%
M+6		1.3%	31%	M+6		1.9%	36%
M+7		0.5%	19%	M+7		0.8%	23%
M+8			13%	M+8			14%
M+9			10%	M+9			9%
M+10			19%	M+10			14%
M+11			28%	M+11			23%
M+12			33%	M+12			27%
M+13			31%	M+13			27%
M+14			25%	M+14			23%
M+15			18%	M+15			14%
M+16			11%	M+16			9%

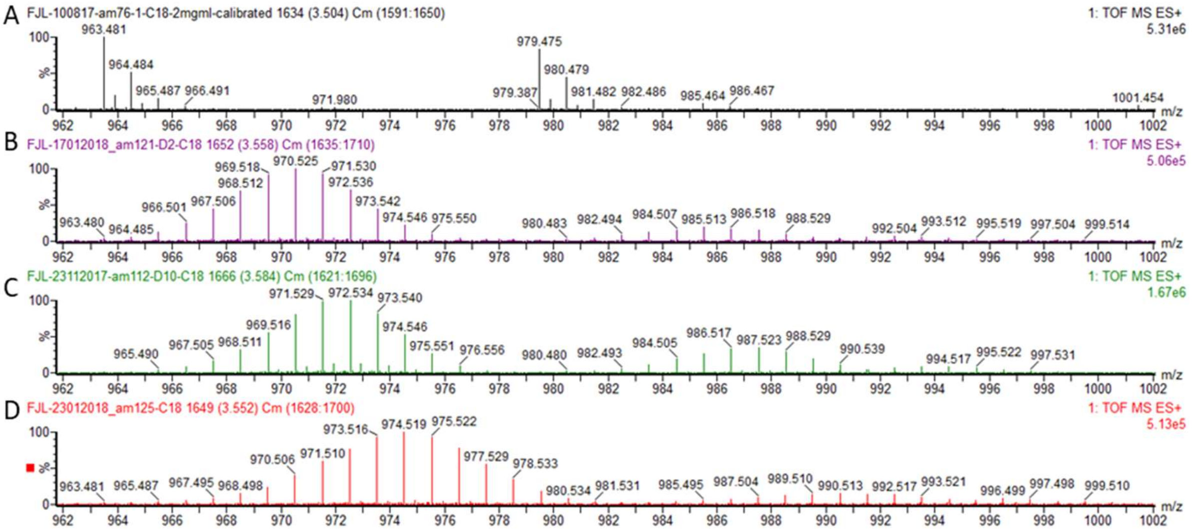

Figure S19. Observed isotope patterns for the 963 and 979 ions for solanimycin isolated from cultures that were fed [$^{2}H_2$]glycine at a concentration of (**A**) 0 mM; (**B**) 2 mM or (**C**) 10 mM or (**D**) were fed [$^{13}C_2$]glycine at a concentration of 2 mM.

Table S3. Abundances of ions related to the solanimycin ions, including ions containing ^{13}C (naturally occurring and due to incorporation of [$^{13}C_2$]glycine) or ^2H (as result of incorporation of universally labelled [$^2H_2$]glycine).

	[$^2H_2$]glycine			[$^{13}C_2$] glycine		[$^2H_2$]glycine			[$^{13}C_2$] glycine
963.48	0 mm	2 mM	10 mM	2 mM	979.477	0 mM	2 mM	10 mM	2 mM
M	100%	4%	1%		M	100%	2%	1%	
M+1	52%	6%	2%	3%	M+1	53%	4%	2%	
M+2	16%	13%	4%	5%	M+2	17%	8%	3%	
M+3	4%	25%	8%	9%	M+3	4%	13%	6%	
M+4	1%	45%	17%	14%	M+4	1%	16%	11%	
M+5		70%	33%	23%	M+5		19%	19%	5%
M+6		91%	55%	38%	M+6		17%	26%	7%
M+7		100%	81%	57%	M+7		15%	33%	9%
M+8		93%	100%	73%	M+8		10%	34%	13%
M+9		70%	100%	88%	M+9		6%	28%	16%
M+10		45%	81%	95%	M+10			19%	18%
M+11		22%	52%	88%	M+11			10%	19%
M+12		10%	26%	74%	M+12				17%
M+13		4%	10%	53%	M+13				16%
M+14			3%	33%	M+14				12%
M+15				17%	M+15				9%
M+16				8%					
M+17				5%					

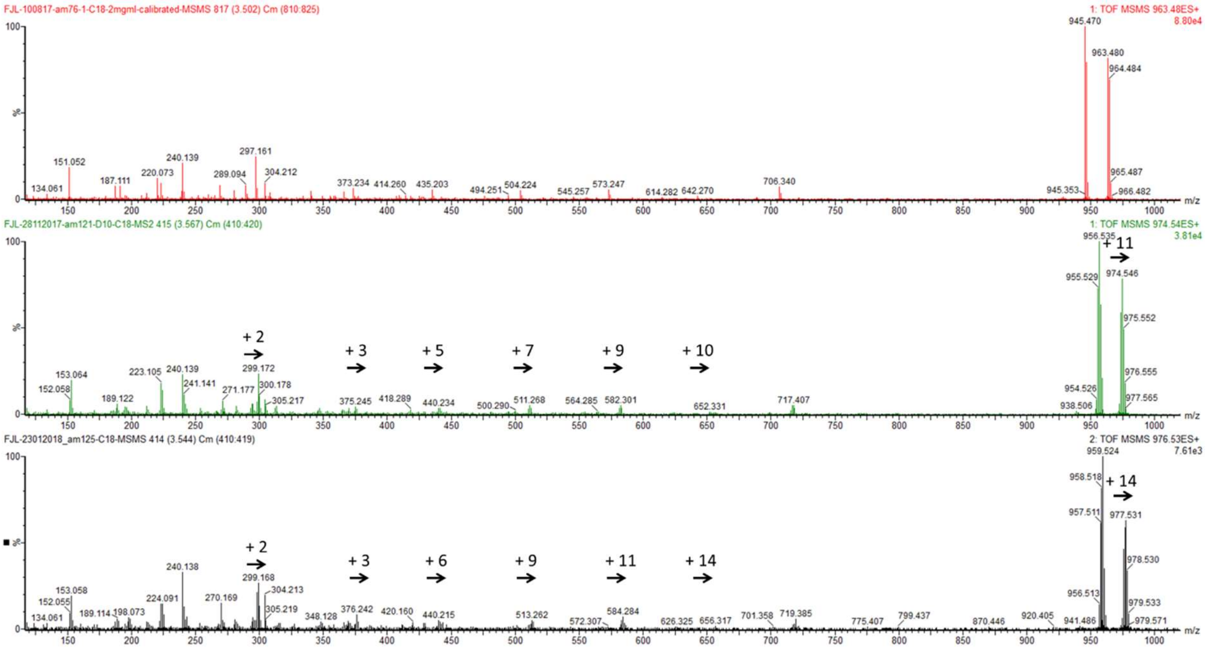

Figure S20. Fragmentation patterns of solanimycin A: (top) unlabelled (*m/z* 963); (middle) after feeding feeding [$^2H_2$]glycine at 10 mM (fragmentation around M+11 ion); (bottom) after feeding [$^{13}C_2$]glycine at 2 mM (fragmentation around M+14 ion). Approximate increases in mass observed for selected fragments are indicated relative to the unlabelled solanimycin A.

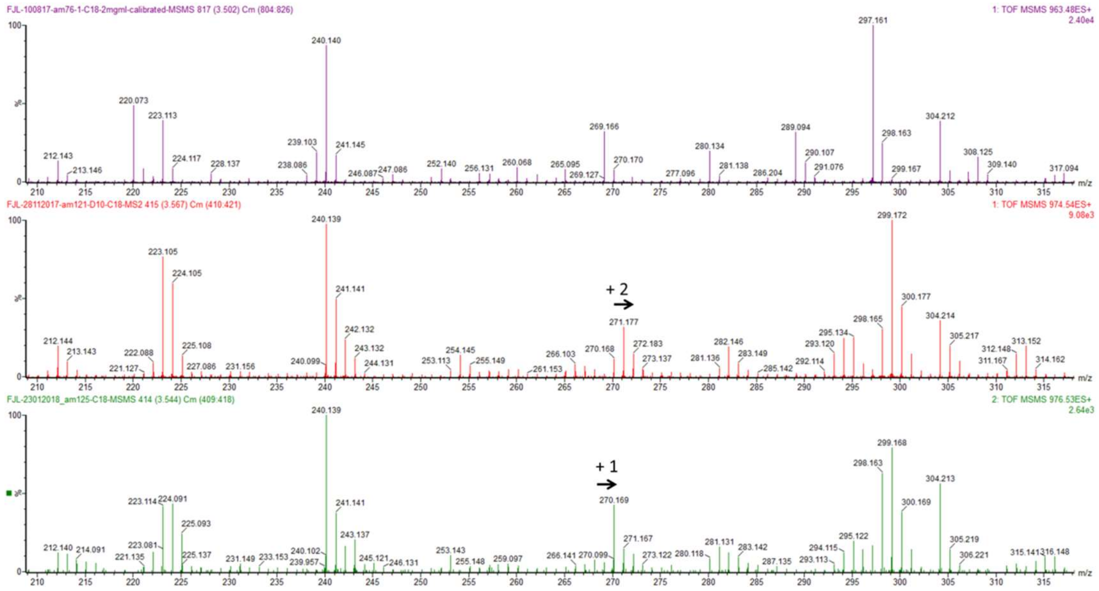

Figure S21. Expansion of the fragmentation patterns for solanimycin A: (top) unlabelled (*m/z* 963); (middle) after feeding feeding [$^2H_2$]glycine at 10 mM (fragmentation around M+11 ion); (bottom) after feeding [$^{13}C_2$]glycine at 2 mM (fragmentation around M+14 ion). Approximate increases in mass observed for one selected fragment are indicated relative to the unlabelled solanimycin A.

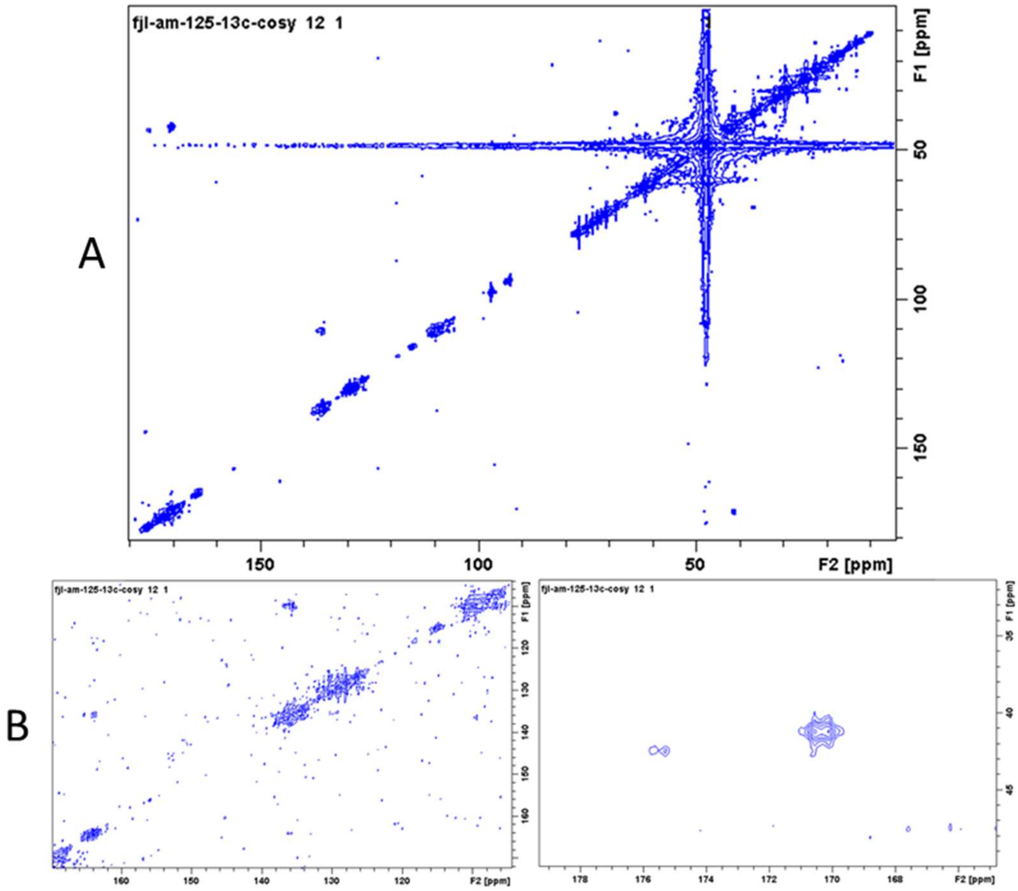

Figure S22. (**A**) ^{13}C-^{13}C COSY NMR spectrum for partially purified solanimycin isolated from cultures fed [$^{13}C_2$]glycine at a concentration of 2 mM. (**B**) Expansions of selected regions of the spectrum.

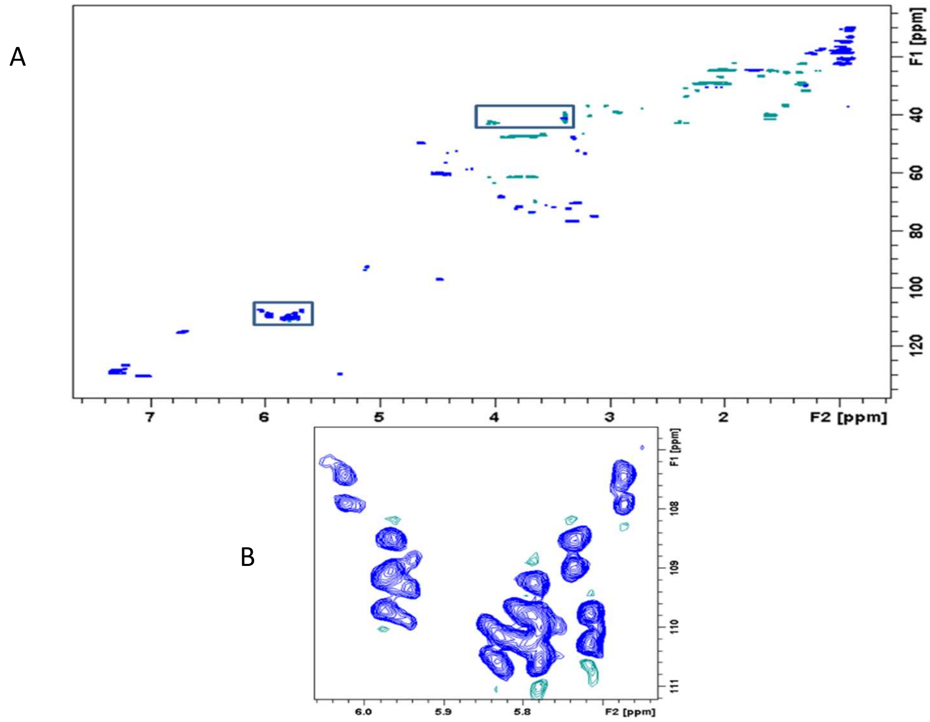

Figure S23. (**A**) HSQC NMR data for partially purified solanimycin isolated from cultures fed [$^{13}C_2$]glycine at a concentration of 2 mM. (**B**) Expansion of a selected region of the spectrum.

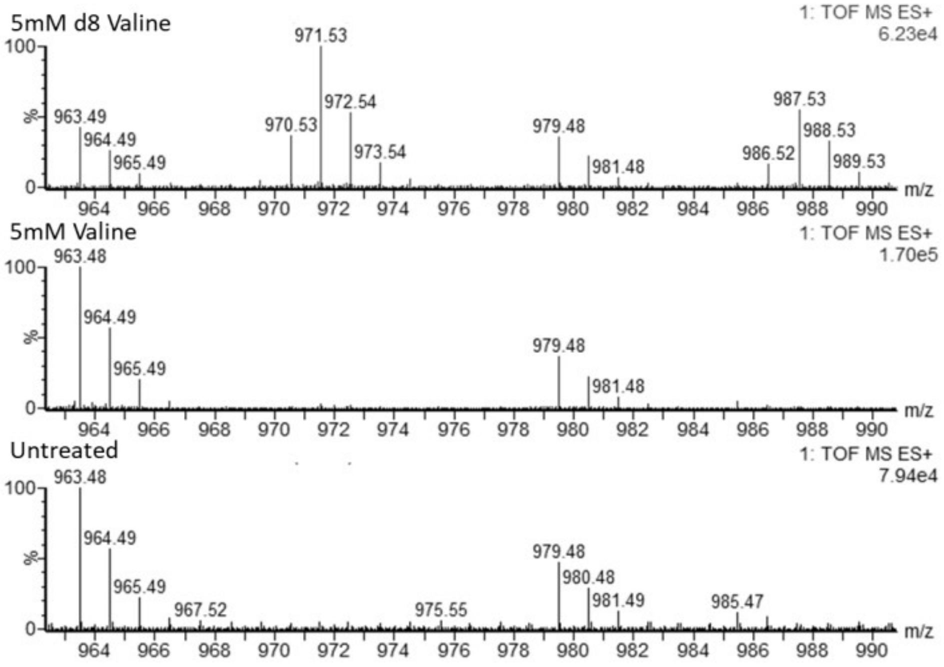

Figure S24: LCMS mass spectra of partially purified solanimycins from cultures fed with: (top) 5 mM d_8-valine; (middle) 5 mM unlabelled valine; (bottom) no additive.

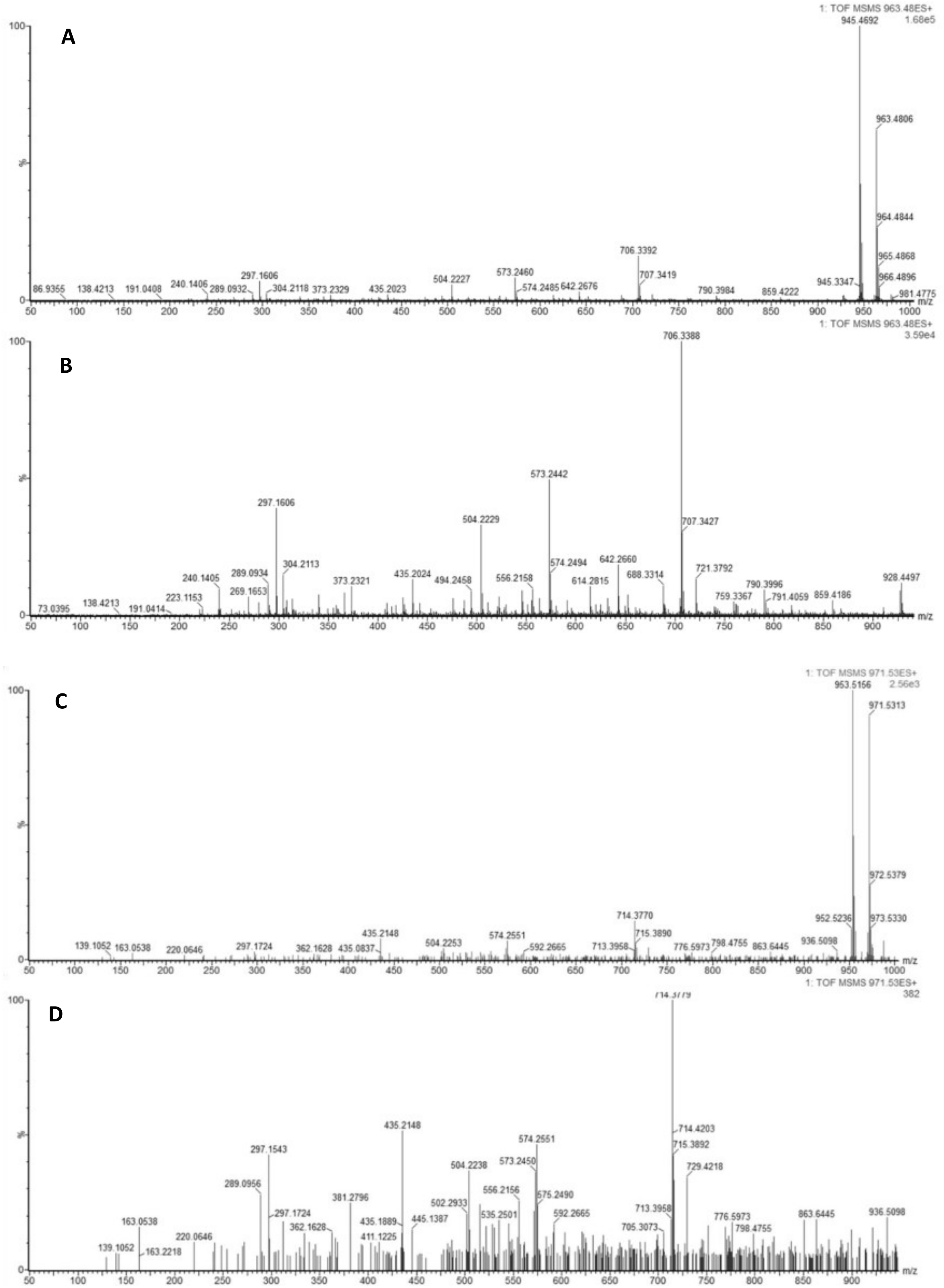

Figure S25: Collision induced fragmentation of solanimycin A with and without feeding of d_8-L-valine. **A**) Whole spectrum, unlabelled solanimycin A (*m/z* 963.48). **B**) Expansion of lower intensity fragments in **A**. **C**) Whole spectrum, d_8-L-valine-labelled solanimycin A (fragmentation centred on *m/z* 971.53). **D**) Expansion of lower intensity fragments in **C**.

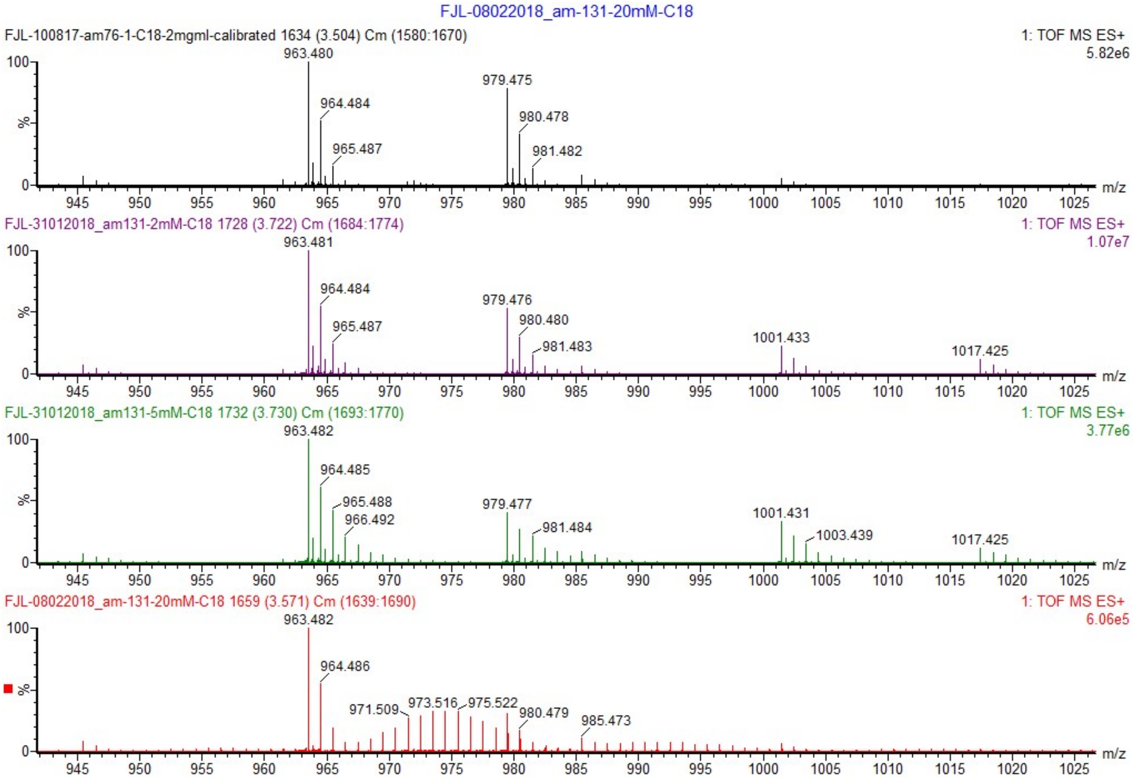

Figure S26. Observed isotope patterns for solanimycins A and B isolated from cultures fed 0, 2, 5 or 20 mM sodium [$^{13}C_2$]acetate (top to bottom).

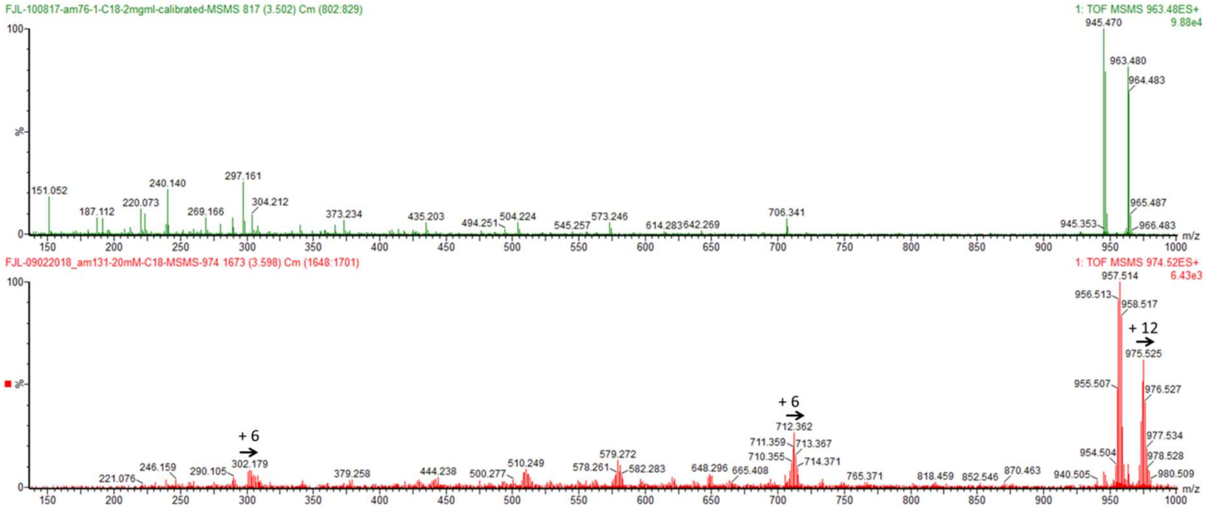

Figure S27. Fragmentation patterns for solanimycin A: (top) unlabelled (*m/z* 963); (bottom) after feeding sodium [$^{13}C_2$]acetate at 20 mM (fragmentation around M+12 ion). Approximate increases in mass observed for selected fragments are indicated relative to the unlabelled solanimycin A.

Table S4. Abundances of isotopically shifted ions related to the solanimycin A (left) or solanimycin B (right) ion after feeding sodium [$^{13}C_2$]acetate at the concentration shown. All abundances are relative to the peak at *m/z* 963.48.

	[$^{13}C_2$]AcONa				[$^{13}C_2$]AcONa		
	2 mM	5 mM	20 mM		2 mM	5 mM	20 mM
963.48 (M)	100%	100%	100%	979.48 (M)	54%	40%	35%
M+1	55%	62%	55%	M+1	30%	27%	20%
M+2	24%	42%	19%	M+2	15%	22%	8%
M+3	9%	21%	7%	M+3	6%	12%	3%
M+4	4%	15%	7%	M+4	4%	9%	2%
M+5	2%	8%	10%	M+5	2%	5%	2%
M+6		6%	16%	M+6		3%	11%
M+7		4%	19%	M+7		1%	7%
M+8		3%	27%	M+8			7%
M+9		2%	29%	M+9			8%
M+10		1%	33%	M+10			8%
M+11			32%	M+11			8%
M+12			33%	M+12			8%
M+13			28%	M+13			8%
M+14			24%	M+14			6%
M+15			19%	M+15			6%
M+16			14%	M+16			6%
M+17			10%	M+17			5%
M+18			7%	M+18			3%
M+19			4%	M+19			3%
M+20			3%	M+20			2%
M+21			2%	M+21			

Expression and Analysis of SolG

Generation of solG-containing Plasmid

solG was amplified by PCR with Phusion polymerase by primers Mat7F and Mat7R containing restriction sites BamHI and HindIII, respectively. An annealing temperature of 65°C and extension time of 3 min gave a single product of the correct size by gel electrophoresis (1% agarose). Thermofisher GeneJET PCR clean-up kit was used to clean up the PCR reaction mixture prior to restriction digestion. Double Bam HI/Hind III digests were performed with New England Biolab (NEB) high fidelity enzymes on pQE80-OriT purified with Thermofisher Genejet plasmid miniprep kit and PCR-amplified *solG*. The DNA was separated from protein via phenol/chloroform extraction. Thermofisher T4 ligase was used to ligate pQE80-OritT and *solG*. The ligation mixture (10 μl) was used directly to transform chemically competent *E. coli* DH5α cells (75 μl) by heat shock at 42°C for 30-60 seconds. Cells were allowed to recover in LB and plated on LB with 100 μg/mL ampicillin. Sequencing was performed by GATC Life Sciences.

Primers used

Mat7F: Forward primer for SolG with a BamHI restriction site. CCCGGATCCATGAGCGATTTCGAACAATT
Mat7R: Reverse primer for SolG with a HindIII restriction site. CCCAAGCTTTCATAATCTCATCTCATCG

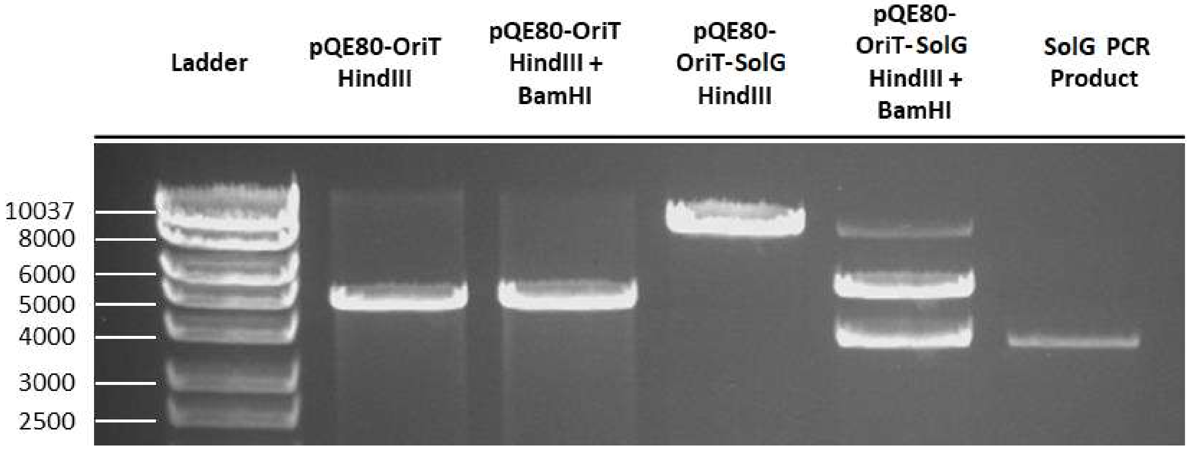

Figure S28. Restriction digest of the pQE80-OriT-(6xHis)-*solG* construct. Initial verification of *E. coli* DH5α transformants was achieved by plasmid extraction and digestion by restriction enzymes HindIII and BamHI. The plasmid extracted after transformation formed a higher molecular weight band by single digest and two lower bands corresponding to linear pQE80-OriT vector and *solG* when double digested.

Expression and Purification of SolG

Protein expression was performed with *E. coli* BL21 (DE3) that had been transformed by heat shock with the pQE80-OriT-*solG* plasmid. 100 mL of culture was inoculated (1%) with 1 mL of an overnight culture of *E. coli* BL21 (DE3) grown at 37°C and then cultured at 37°C, reaching an A_{600} of 0.6-0.7 after 3.5 h. Cultures were then induced with 100 μM isopropyl-β-D-thiogalactoside (IPTG) and incubated at 16°C for a further 18 h for protein expression. Cells were pelleted via centrifugation at 4 °C and the supernatant discarded. The cell pellet was resuspended in 10 mL of buffer A (50 mM Tris-HCl, 300 mM NaCl, 20 mM imidazole, pH 8) supplemented with 1 mg/mL lysozyme. Cells were lysed by sonication comprised of five pulses of 20 s separated by 30 s rest periods. Centrifugation at 4°C gave soluble and insoluble fractions. The soluble fraction was applied to a column containing Qiagen Ni-NTA Agarose (1 mL per 4 mL of cleared lysate) equilibrated with 10 mL buffer A and the flow through collected, the column was washed with buffer A in two steps (10 mL each) and then SolG was eluted with 10 mL of buffer B (50 mM Tris-HCl, 300 mM NaCl, 250 mM imidazole, 0.5 mM tris(2-carboxyethyl) phosphine (TCEP), pH 8) and 2 mL fractions collected. After SDS-PAGE analysis, fractions 1 and 2 were pooled and dialysed against buffer C (50 mM Tris-HCl, 300 mM NaCl, 0.5 mM TCEP, pH 8) overnight. The dialysate was kept and used as blanks or to dilute the samples as necessary in downstream applications.

Sodium Dodecyl Sulfate-Polyacrylamide Gel Electrophoresis (SDS-PAGE)

Polyacrylamide (15%) Bis-Tris gels were routinely run at a constant 100 V at 4°C for 5 hours with a running buffer comprised of 0.05% SDS, 600 mM glycine and 25 mM Tris and sample loading buffer with a final concentration of 62.5 mM Tris (pH 6.8), 2% SDS, 0.01% bromophenol blue, and 350 mM DTT. Gels were stained with a solution containing methanol:acetic acid:water (1:2:7 by volume) and 1 g/L Coomassie R-250. After a suitable time, the gel was de-stained with water overnight. Western blotting of gels was achieved by transfer of protein to a PVDF membrane activated with methanol in transfer buffer comprised of 560 mM glycine, 25 mM Tris and 15% w/v methanol. After transfer at a

constant 250 mA for 90 min, the membrane was blocked with 50 g/L powdered milk in phosphate-buffered saline supplemented with 0.1% Tween 20 (PBST) for 1 h. The Anti-6xHis primary antibody (mouse) was then added and left on a rocking platform for 1 h before being washed 4x with PBST. The membrane was then then incubated with the anti-mouse horseradish peroxidase-conjugated secondary antibody in 30 mL blocking solution for 30 min before being washed 4x with PBST. Visualization was achieved photometrically with Biorad ECL substrates for horseradish peroxidase and standard X-ray film. Unless specified, films were exposed for 10 min before developing.

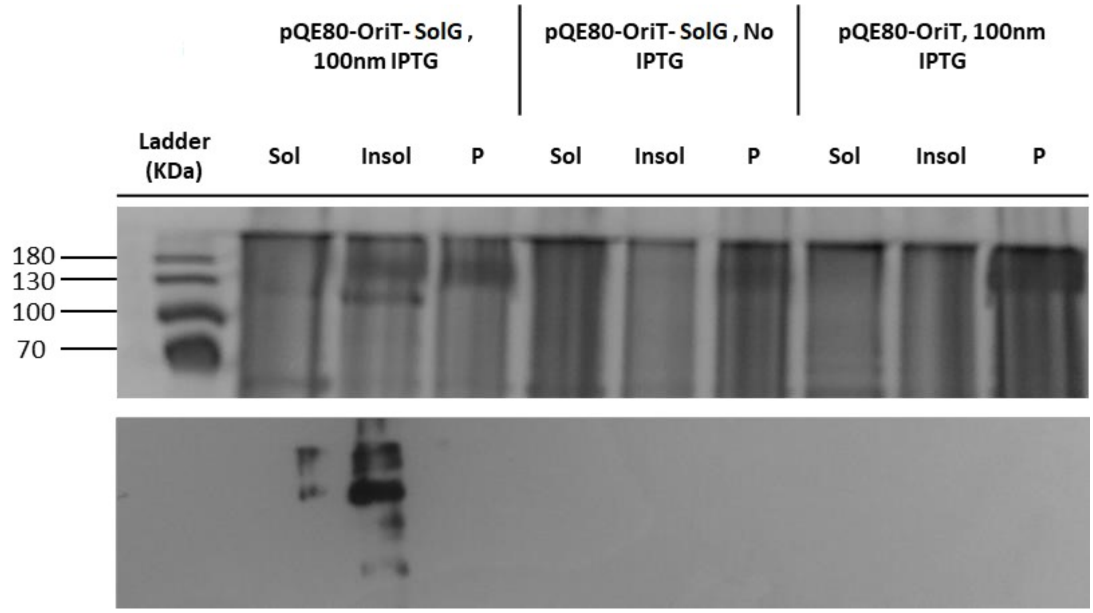

Figure S29. Analysis of soluble fractions (Sol), insoluble fractions (Insol) and cells pelleted at end of the protein expression period (P) by SDS-PAGE. Cells had been diluted with buffer A (see methods) to the optical density of the culture they were derived from. No significance differences in growth were seen between cultures. Top, Coomassie-stained gel. Bottom, Western blot of gel after transfer to a PVDF membrane (see methods).

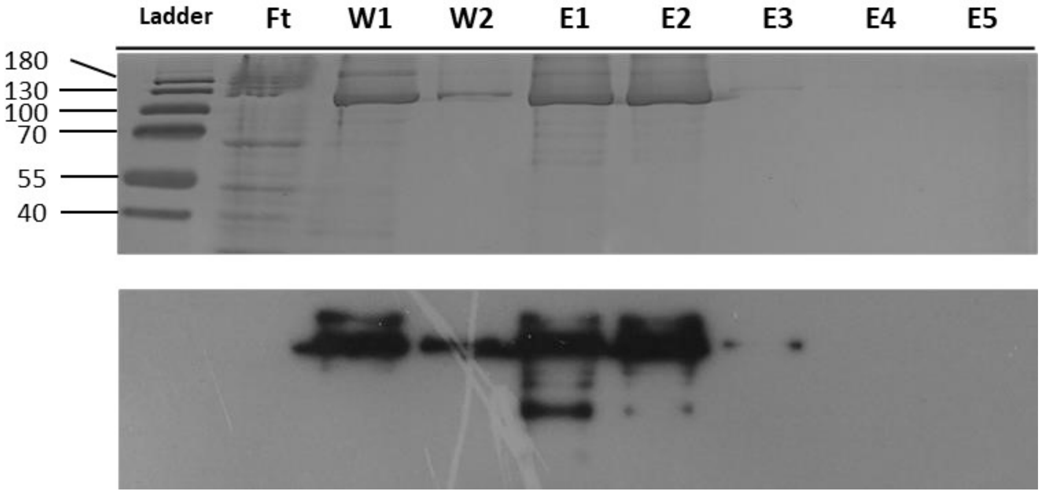

Figure S30. SDS PAGE analysis of SolG from the soluble fraction after purification by nickel-NTA affinity chromatography. Ft = flow-through; W1-2 = washes; E1-5 = eluted fractions. Top: Coomassie stained gel. Bottom: Western blot of gel after transfer to a PVDF membrane.

Blue Native PAGE

Blue native PAGE (BN-PAGE) 3-12% Bis-Tris gradient protein gels were supplied by Invitrogen. 18.75 μL of sample was made up to a total volume of 25 μL with 6.25 μL of loading buffer comprised of 50 mM Bis-Tris, 50 mM NaCl, 10% glycerol, 0.001% Ponceau S, and 0.6N HCl. Gels were run for 120 min at constant 150 V with an anode buffer consisting of 50 mM Bis-Tris and 50 mM Tricine buffered to pH 6.8 and a cathode buffer consisting of the above supplemented with 0.04% Coomassie G-250 (w/v). Gels were stained with a fixing solution containing methanol:acetic acid:water (4:1:5), a stain solution of methanol:acetic acid:water (3:1:6) and 0.02% Coomassie G-250, and a destaining solution of 8% acetic acid. Western blotting was performed as for SDS-PAGE analysis.

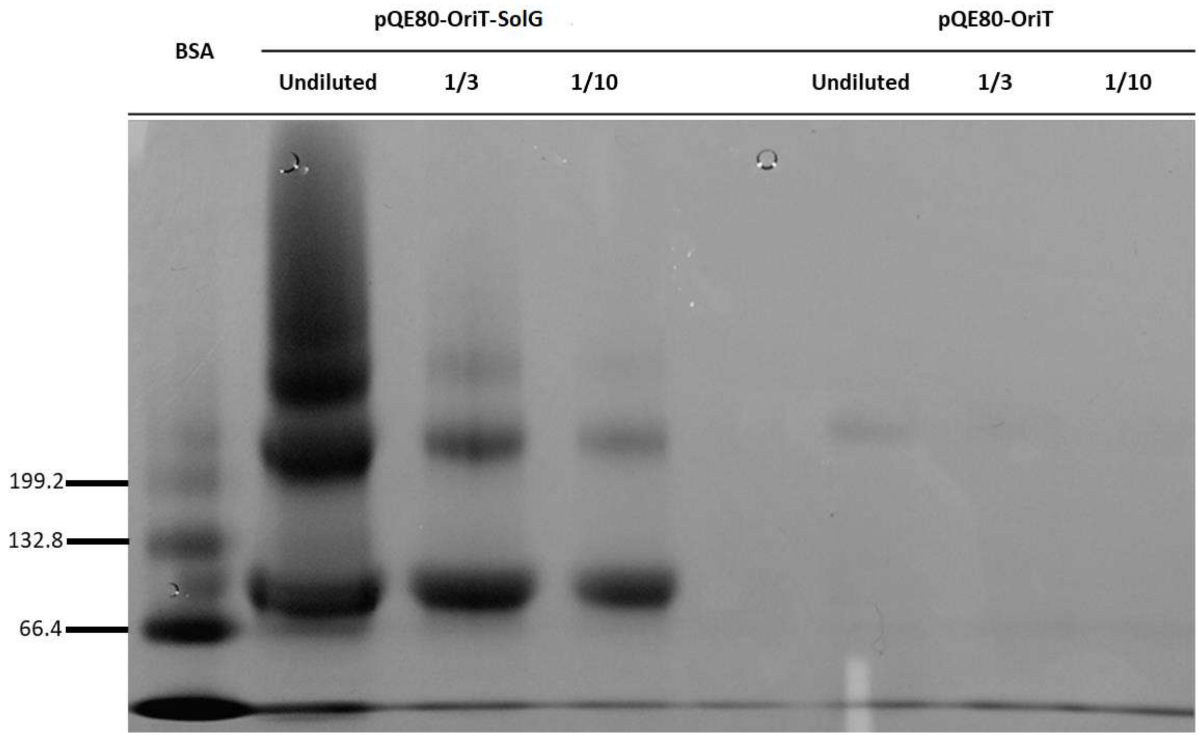

Figure S31. Coomassie stained Blue Native-PAGE of purified SolG and purified product from a culture of *D. solani* containing empty vector. Uniform running was achieved by loading of all wells with loading buffer (bottom band). A BSA standard of 1 mg/mL was used as a ladder as BSA is known to form dimer (132.8 kDa) and trimers (199.2 kDa) in solution at high concentrations. The formation of higher order oligomers at higher concentrations of SolG may be due to non-specific aggregation and/or disufide bond formation (*e.g.* between the thiolation domains) of monomers.

Analytical Ultracentrifugation

Analytical ultracentrifugation (AUC) was performed (in collaboration with Dr Katherine Stott of the University of Cambridge Department of Biochemistry Biophysics Facility) on a Beckman Optima XL-I equipped with interference and absorbance optics, with the latter taking readings at 275 nm. 400 μL of SolG solution was placed in one (sample) compartment of an Epon double-sector centrepiece and 400 μL of dialysate placed in the other (reference) compartment. The samples were centrifuged at 20°C in an An-60-Ti rotor at 40,000 rpm. Scans were acquired using both systems at time intervals of 3 min. The calculated partial, specific, and buffer viscosity and density were obtained using the

public domain program SEDNTERP developed by Hayes, Laue, and Philo[1] (http://www.jphilo.mailway.com). Analysis was performed using sedfit[2]. Samples run consisted of undiluted purified protein at ~1 mg/ml, a 1:3 dilution, and a 1:10 dilution. Samples were diluted with dialysate to maintain sample homogeneity.

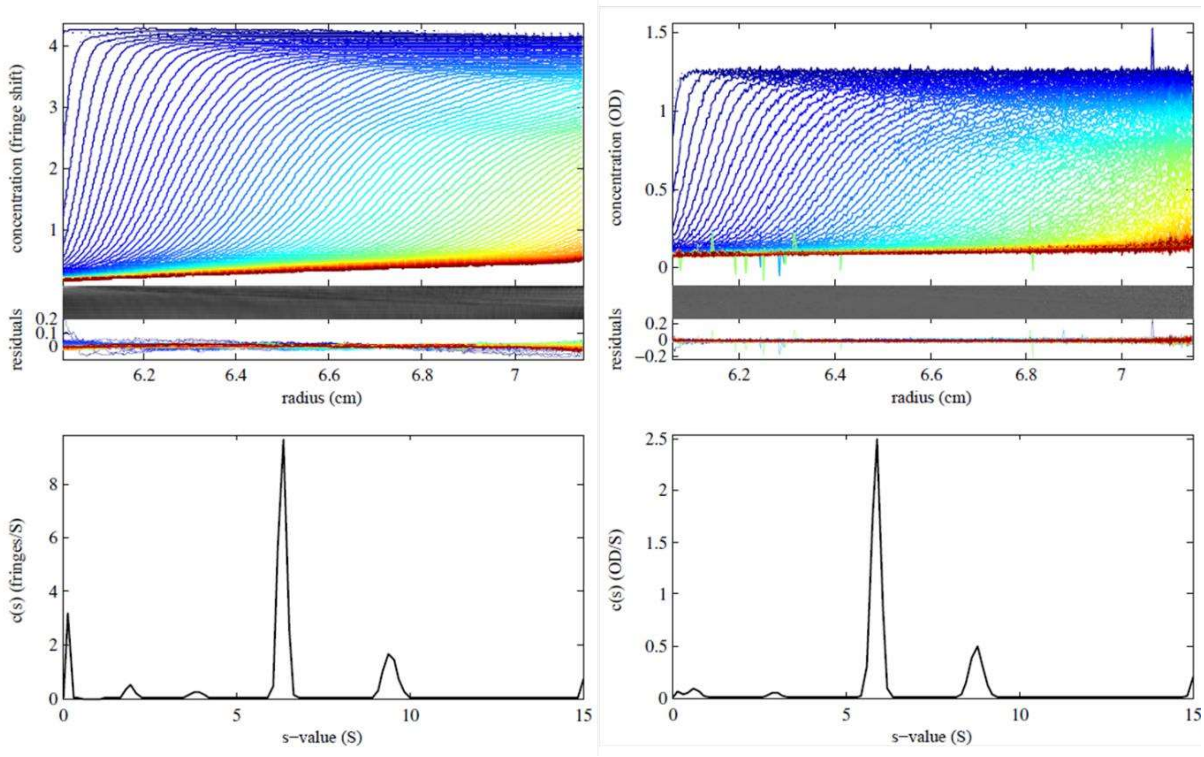

Figure S32. Analytical ultracentrifugation monitored by interference (left) and by UV detection at 275 nm (right). Top: boundary distributions across sample cell as a function of time (blue to red as time went on). Middle: 2-dimensional representation of residuals of data fitting (uniform grey represents randomly distributed residuals arises from good data fitting). Bottom: sedimentation distribution plot shows predominantly monomer (S ≈ 6.3) and dimer (S ≈ 9.4)

Non-Denaturing Mass Spectrometry

Protein samples were concentrated to *ca*. 33 μM and exchanged into 200 mM aq. ammonium acetate by spin column. Non-denaturing mass spectroscopy was performed on a Waters Synapt high definition mass spectrometry system equipped with a nanoESI electrospray source with a chip nozzle voltage of 1.71 kV and a cone voltage of 89 V.

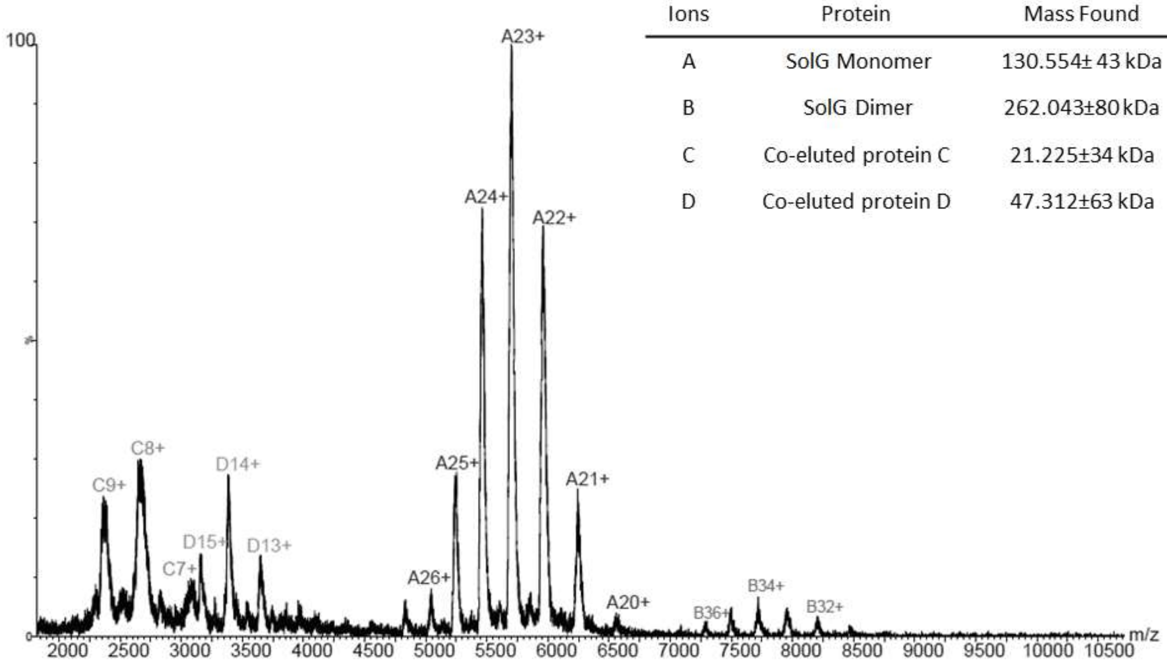

Ions	Protein	Mass Found
A	SolG Monomer	130.554± 43 kDa
B	SolG Dimer	262.043±80 kDa
C	Co-eluted protein C	21.225±34 kDa
D	Co-eluted protein D	47.312±63 kDa

Figure S33. Non-denaturing mass-spectrometry of SolG.

Hydrolysis of solanimycin

Semipurified solanimycin in water was acidified to 2 M with concentrated hydrochloric acid and heated under reflux for 4 h and then left to cool to room temperature. 100 μl of the reaction mixture was diluted with 100 μl of acetonitrile and then analysed by UHPLC/MS eluting with a gradient of 5 to 95% acetonitrile over 5.5 min, then 95% acetonitrile in water for 2 min, then 5% acetonitrile in water for 30 s. Two peaks corresponding to the mass of the C-terminal amine ($M+H^+$ observed 304.2113, calc. 304.2119) and its N-pyruvyl derivative ($M+H^+$ observed 374.2205, calc. 374.2173) were observed at 1.85 min and 3.48 min, respectively. These peaks were not present in the equivalent hydrolysate of the extract from the *sol*$^-$ strain, nor in the semipurified solanimycin before hydrolysis.

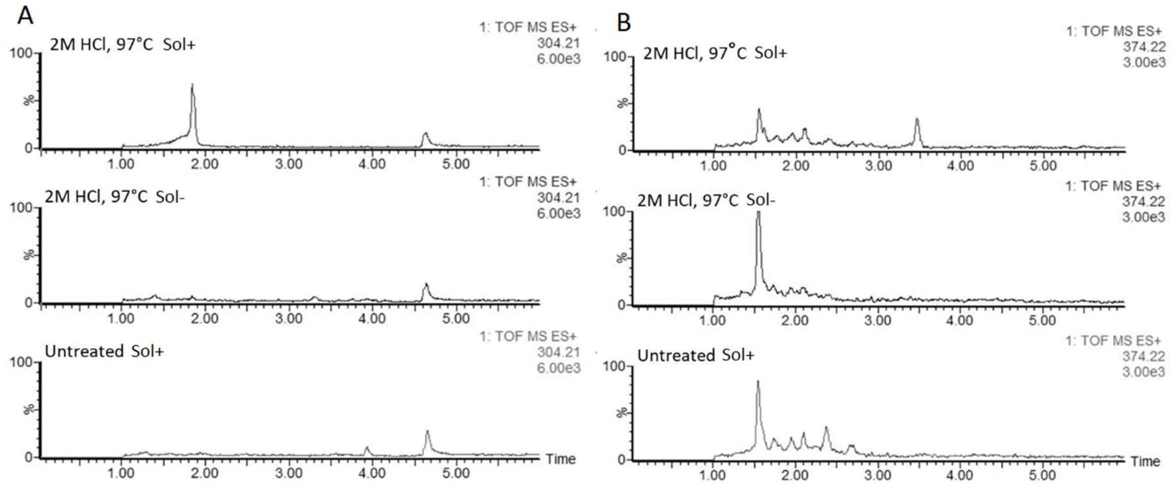

Figure S34. Extracted ion chromatograms at (A) *m/z* 304.21 and (B) *m/z* 374.22 of (top) semipurified solanimycin after hydrolysis, (middle) extract from *sol⁻* strain after hydrolysis, (bottom) semipurified solanimycin before hydrolysis. *M/z* 304.21 corresponds to the M+H$^+$ ion of the C-terminal amine (*e.g.* **10**) and *m/z* 374.22 corresponds to the M+H$^+$ ion of its N-pyruvyl derivative.

References

1. Lebowitz J, Lewis MS, Schuck P. Modern analytical ultracentrifugation in protein science: A tutorial review. *Protein Sci.* 2009; 11 (9): 2067-2079. doi:10.1110/ps.0207702

2. Schuck P. Analytical ultracentrifugation as a tool for studying protein interactions. *Biophys. Rev.* 2013; 5 (2): 159. doi:10.1007/S12551-013-0106-2
